# Mediation Mendelian randomisation study on the effects of shift work on coronary heart disease and traditional risk factors via gut microbiota

**DOI:** 10.7189/jogh.14.04110

**Published:** 2024-05-28

**Authors:** Yun Zhang, Lingyun Zhao, Yifan Jia, Xin Zhang, Yueying Han, Ping Lu, Huijuan Yuan

**Affiliations:** Department of Endocrinology, Henan Provincial People’s Hospital & Zhengzhou University People’s Hospital & People’s Hospital of Henan University, Zhengzhou City, Henan Province, China

## Abstract

**Background:**

Epidemiological evidence suggests that there is an increased risk of coronary heart disease (CHD) related to jobs involving shift work (JSW), but the causality of and mechanism underlying such a relationship remain unclear. Therefore, we aimed to explore the relationship between JSW and CHD, investigating both causality and potential mediating factors.

**Methods:**

We performed univariate, multivariate, and mediation Mendelian randomisation (MR) analyses using data from large genome-wide association studies focussed on JSW and CHD, as well as data on some CHD risk factors (type 2 diabetes, hypertension, obesity, and lipids measurement) and 196 gut microbiota taxa. Single-nucleotide polymorphisms significantly associated with JSW acted as instrument variables. We used inverse-variance weighting as the primary method of analysis.

**Results:**

Bidirectional MR analysis indicated a robust effect of JSW on increased CHD risk; however, the existence of CHD did not affect the choice of JSW. We identified a mediating effects of type 2 diabetes and hypertension in this relationship, accounting for 11.89% and 14.80% of the total effect of JSW on CHD, respectively. JSW were also causally associated with the risk of type 2 diabetes and hypertension and had an effect on nine microbial taxa. The mediating influence of the *Eubacterium brachy* group at the genus level explained 16.64% of the total effect of JSW on hypertension. We found limited evidence for the causal effect of JSW on obesity and lipids measurements.

**Conclusions:**

Our findings suggest a causal effect of JSW on CHD, diabetes, and hypertension. We also found evidence for a significant connection between JSW and alterations in the gut microbiota. Considering that certain microbial taxa mediated the effect of JSW on hypertension risk, targeting gut microbiota through therapeutics could potentially mitigate high risks of hypertension and CHD associated with JSW.

Owing to increased work pressure and the 24-hour/d production cycle in modern society, jobs involving shift work (JSW) have become more common over the past decade. Approximately 19–25% of workers in most countries engage in JSW, especially night shifts [1–3]. Such jobs are among the most common external factors underlying disorganised circadian rhythms, which endogenously regulate behavioural and physiological activities over a 24-hour cycle and are crucial for maintaining health [4].

Numerous studies have linked JSW to an increased risk of coronary heart disease (CHD) and CHD-related mortality, even after adjusting for risk factors. For example, a UK cohort study followed up 276 009 participants without CHD at baseline for a median 10.4 years and found a 1.22-fold increased CHD risk in those with >10 years of night shifts [5]. A meta-analysis of 21 studies also supports this relationship [6]. Moreover, JSW was also found to be associated with CHD risk factors, with studies of American and Danish nurses showing a higher risk of type 2 diabetes (T2DM) in rotating night shift workers [7,8] and several cohort studies finding a positive effect of JSW on blood pressure and obesity [9,10].

However, existing studies on the relationship between JSW and CHD and its risk factors have been observational in design, with conflicting results. For example, Ramin et al. [11] reported a link between the risk of obesity and the number of night shifts per month; conversely, a cohort study of Norwegian nurses found no association between change in body mass index and number of night shifts per year [12]. Moreover, a study in Swedish seafarers showed no evidence of increased cardiovascular mortality [13]. This inconsistency in the findings of traditional observational studies may result from factors such as reverse causation, selection bias, and environmental confounders, which are inherent to this design. They may also arise from differences in study cohort size or in the definition of JSW. Furthermore, observational studies cannot establish causality or identify the mechanisms underlying the associations.

In turn, Mendelian randomisation (MR) allows the causal effects of disease exposure to be evaluated by integrating genomic data into conventional observational studies [14,15], thereby minimising reverse causality and environmental bias by relying on genetic variants that are passed down randomly at conception. We thus investigated the causal link between JSW and CHD via univariate, multivariate, and mediation MR analyses. We also explored the mediating effects of CHD-related risk factors and gut microbiota in this relationship.

## METHODS

### Study design

**Figure 1:**
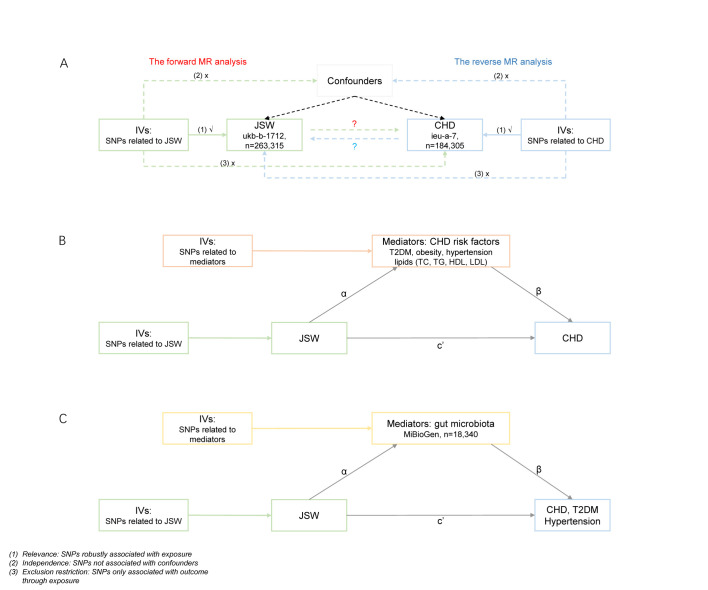
Flowchart of the MR analysis. **Panel A.** A bidirectional MR analysis to assess the causal crosstalk between JSW and CHD. **Panel B.** A two-step mediation MR analysis to assess the mediation effect of CHD risk factors in the association between JSW and CHD. **Panel C.** A two-step mediation MR analysis to assess the mediation effect of gut microbiota in the association between JSW with CHD and CHD risk factors. CHD – coronary heart disease, HDL – high-density lipoprotein cholesterol, IV – instrumental variable, JSW – jobs involving shift work, LDL – low-density lipoprotein cholesterol, MR – Mendelian randomisation; SNP – single-nucleotide polymorphism, TC – total cholesterol, T2DM – type 2 diabetes, TG – triglyceride.

We used univariate, multivariate, and mediation MR analyses to explore the bidirectional causal association between JSW and CHD; the mediation effect of traditional CHD risk factors (including obesity; T2DM; hypertension; and levels of total cholesterol, high-density lipoprotein cholesterol (HDL), low-density lipoprotein cholesterol (LDL), and triglycerides); and the mediation effect of gut microbiota (**Figure 1**). We followed the STROBE-MR guidelines [16,17] in conducting the study and reporting our findings (Table S1 in the **Online Supplementary Document**).

### Data sources

We used summary-level statistics from genome-wide association studies (GWASs) for our MR analysis. Details of each cohort are presented in Table S2 in the **Online Supplementary Document**.

We retrieved GWAS data associated with JSW from the UK Biobank, which covers approximately 9 851 867 SNPs from 263 315 European individuals [18]. The Biobank collected relevant information, including JSW status, through a self-reported touchscreen questionnaire completed by participants (all of whom provided written informed consent).

We obtained GWAS summary data associated with CHD from the CARDIoGRAMplusC4D project. The CARDIoGRAMplusC4D 1000 Genomes-based GWAS is a meta-analysis of GWASs that analysed 9.4 million variants and involved 60 801 CHD cases and 123 504 controls, mainly from European, South Asian, and East Asian populations [19]. The diagnosis of CHD was done based on the International Classification of Diseases, Tenth Revision (ICD-10) criteria (I20, I21, and I22).

We extracted GWAS data on obesity, T2DM, and hypertension from the FinnGen project, a large public-private partnership that collects and analyses genomics and health data from 500 000 Finnish Biobank participants. We also obtained GWAS data on lipid measurements from a joint analysis of GWAS associations performed by the Center for Statistical Genetics, which examined 188 577 individuals [20].

Lastly, we used the summary-level GWAS data related to the human gut microbiota from the MiBioGen study, a multi-ethnic GWAS meta-analysis involving 18 340 participants from 24 cohorts. The study, which focussed on gut microbiota, was conducted by the MiBioGen team. They assessed the microbiota composition of human faecal samples using 16S rRNA sequencing and classified them into five categories: 9 phyla, 16 classes, 20 orders, 35 families, and 131 genera. We excluded twelve unknown genera and three unknown families from this sample [21,22].

### Selection of eligible instrumental variables

We identified single-nucleotide polymorphisms (SNPs) as eligible instrumental variables (IVs) based on the following criteria:

− SNPs must be robustly related to the levels of exposure. We chose a genome-wide significance threshold of *P* < 1 × 10^5^ for JSW and gut microbiota, and *P* < 5 × 10^8^ for CHD and risk factors, as in previous studies [23,24];
− The SNPs must be independent, defined as having a low linkage disequilibrium threshold (r^2^ < 0.001, kb = 10 000) in the European population [23];
− To ensure that SNPs are related to outcomes only by way of exposure, they must not be significantly associated with the outcome or confounders.

We used the PhenoScanner tool to assess the effects of confounders (age, sex, smoking and diet) and to exclude variables accordingly [25]. We used MR pleiotropy residual sum and outlier (MR-PRESSO) to identify possible outliers, which we then excluded from further analysis. We also computed the F-statistic for all SNPs and selected those with high F-statistics (>10) as IVs with high explanatory value.

### Statistical methods and sensitivity analysis

For the univariate MR analysis, we used the inverse-variance-weighted (IVW) mode as the primary method for evaluating the causative links between exposure and outcome, combined with simple-mode and weighted-median analyses for supplementary data. We further assessed for heterogeneity among the causal effects using Cochran’s Q test, explored horizontal pleiotropy through MR-Egger regression and the global test of MR-PRESSO, and conducted sensitivity analyses through the leave-one-out approach.

We performed a two-step Mendelian MR analysis to estimate the mediating effect of CHD-related risk factors and gut microbiota in the association between JSW and CHD. Specifically, we first used a univariate MR analysis to identify the causal effect of JSW on CHD risk factors and gut microbiota (β1). Here, we selected CHD risk factors and gut microbiota taxa significantly associated with JSW as potential mediators. We then conducted a multivariable MR analysis to estimate the effect of each mediator on the risk of CHD, adjusting for the genetic effect of JSW (β2), and to estimate the direct effect of JSW on CHD, adjusting for each mediator (β3). We calculated the indirect effect as β1 × β2, the total effect as β1 ×  β2 + β3, and the proportion of mediation effect as β1 × β2 / (β1 × β2 + β3). We used a fixed-effect model of IVW as the major method for the multivariable MR analysis, unless heterogeneity existed (*P* < 0.05), in which case we employed a random-effect IVW model.

We performed all analyses using R, version 4.3.1 (R Core Team, Vienna, Austria), with the ‘TwoSampleMR’ and ‘MendelianRandomization’ package being used for the univariate and multivariate MR-related analyses, respectively. We presented the results of our MR analysis as odds ratio (OR) and 95% confidence intervals (CIs). We set the threshold for statistical significance at *P* < 0.05 and applied a false discovery rate (FDR) correction. Here, a *P*-value of <0.05 without correction (*P*_FDR_≥0.05) indicated a suggestive association, while a *P*-value of <0.05 with *P*_FDR_<0.05 indicated a significant causal association.

### Ethical approval

We used summary-level data from GWASs for the MR analysis which were available from public databases; thus, we required no ethical approval.

## RESULTS

### Bidirectional causal effect of JSW on CHD

After removing SNPs directly associated with confounders and outcomes, we identified 43 SNPs as potential IVs for JSW based on exposure criteria (Table S3 in the **Online Supplementary Document**). All SNPs selected as IVs exhibited high explanatory value, with F-statistics of 19–27, indicating a lower possibility of weak instrumental variable bias.

**Figure 2:**
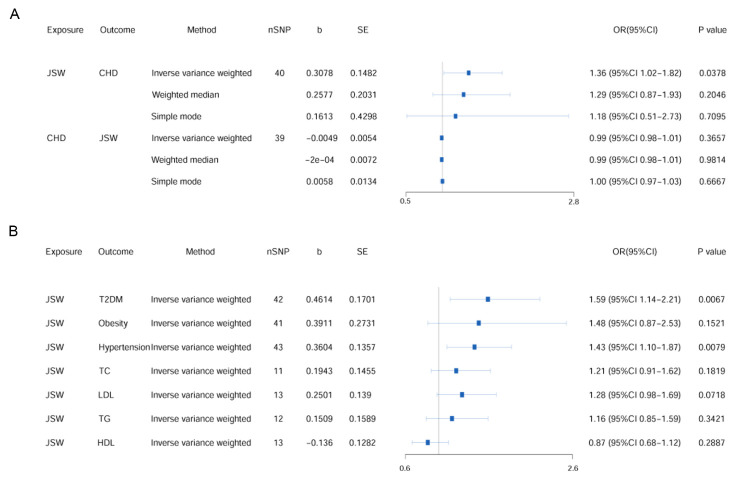
Causal effect of JSW on CHD and CHD-related risk factors. **Panel A.** Bidirectional causal crosstalk between JSW and CHD. **Panel B.** Causal effect of JSW on traditional risk factors related to CHD. CHD – coronary heart disease, HDL – high-density lipoprotein cholesterol, JSW – jobs involving shift work, LDL – low-density lipoprotein cholesterol, nSNP – numbers of single-nucleotide polymorphisms, TC – total cholesterol, TG – triglyceride, T2DM – type 2 diabetes.

Using the IVW method, we identified a causal effect of JSW on increasing the risk of CHD (OR = 1.36; 95% CI = 1.02–1.82, *P* = 0.04) (**Figure 2**, Panel A; Table S4 in the **Online Supplementary Document**). The results from the weighted-median and simple-mode analyses supported the nature of this relationship, with the same direction. Cochran’s Q test for IVW showed no heterogeneity (Q = 25.8777, *P* = 0.95), while the MR-Egger intercept term (intercept = −0.0030, *P* = 0.66) and the MR-PRESSO global test (*P* = 0.94) found no pleiotropy or outliers. We observed no significant influence of SNPs impacting causality (Figure S1 in the **Online Supplementary Document**.

To explore if the existence of CHD had an effect on the choice of JSW, we also performed a reverse MR analysis with CHD as exposure and JSW as outcome. We obtained no significant results in the IVW, weighted median, or simple-mode analyses.

### Causal effect of JSW on CHD mediated by traditional risk factors

Given that JSW have been reported to be associated with traditional risk factors related to CHD, we assessed whether the causal effect of JSW on CHD risk was mediated by traditional CHD risk factors (**Figure 2**, Panel B; Table S4 in the **Online Supplementary Document**). The univariate MR analysis showed that an increase of one standard deviation in JSW was causally associated with a 1.59-fold increased risk of T2DM (95% CI = 1.14–2.21, *P* = 0.01) and a 1.43-fold increased risk of hypertension (95% CI = 1.10–1.87, *P* = 0.01), both remaining significant even after FDR correction (*P*_FDR_ = 0.03). We observed no significant associations between JSW and obesity or lipid measurements (total cholesterol, triglyceride, HDL, and LDL).

**Figure 3:**
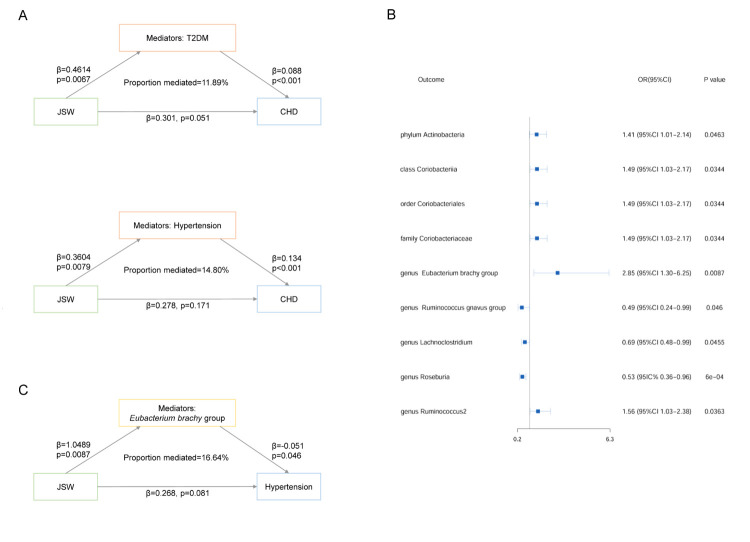
Mediating effect of CHD risk factors and gut microbiota on JSW and CHD risk association. **Panel A.** MR-estimated mediating effects of T2DM and hypertension in the association of JSW and CHD risk, respectively. **Panel B.** MR-estimated effects of JSW on each gut microbiota taxon. **Panel C.** MR-estimated mediating effect of *Eubacterium brachy* group in the association of JSW and CHD risk. CHD – coronary heart disease, CI – confidence interval, JSW – jobs involving shift work, MR – Mendelian randomisation, nSNP – numbers of single-nucleotide polymorphisms, T2DM – type 2 diabetes.

We then separately adjusted the causal associations between T2DM or hypertension and CHD risk for the effect of JSW in the multivariate MR analysis (**Figure 3**, Panel A; Table S5 in the **Online Supplementary Document**). We found that T2DM was significantly associated with an increased risk of CHD after adjusting for JSW (β2 = 0.088; 95% CI = 0.041–0.135, *P* < 0.01), indicating a complete mediating effect (β for indirect effect = 0.0406; 95% CI = 0.0090–0.0830). As we detected heterogeneity for hypertension (*P* = 0.04), we employed a random-effect IVW model. We found a significant indirect effect of hypertension in the causal association between JSW and CHD (β for indirect effect = 0.0483, 95% CI = 0.01–0.09). T2DM and hypertension accounted for 11.89% and 14.80% of the overall mediating effect, respectively. The multivariate MR analysis showed no horizontal pleiotropy.

### Causal effect of JSW on CHD and CHD risk factors mediated by gut microbiota

Both JSW and CHD have been reported to be associated with the dysbiosis of gut microbiota. We therefore explored the mediating effect of the gut microbiota in the association between JSW and CHD risk.

Through the univariate MR analysis of the associations between genetically predicted JSW and each of the 196 gut microbiota taxa (**Figure 3**, Panel B; Table S6 in the **Online Supplementary Document**), we identified nine bacterial taxa influenced by JSW. Within the phylum Actinobacteria, the abundance of Actinobacteria at the phylum level (ID 400), Coriobacteriia at the class level (ID 809), Coriobacteriales at the order level (ID 810), and Coriobacteriaceae at the family level (ID 811) was increased in workers with JSW. Within the phylum Bacillota, we identified suggestive effects of JSW on the abundance of five genera, with one genus showing an increase in abundance (*Eubacterium brachy* group (ID 11296)) and four showing a decrease in abundance (*Ruminococcus gnavus* group (ID 14376), *Lachnoclostridium* (ID 11308), *Roseburia* (ID 2012), and *Ruminococcus*2 (ID 11374)). All five genera belong to the order Eubacteriales. None of the effects of JSW on bacterial taxa remained significant after FDR correction. We found no pleiotropy or outliers and observed no significant influence of SNPs impacting causality (Figure S2 and Table S7 in the **Online Supplementary Document**).

The causal effects of the nine taxa on CHD risk were then adjusted for JSW in the multivariate MR analysis (Table S5 in the **Online Supplementary Document**). The proportion of the mediation effect that could be explained by each taxon was 3.94–10.96%. However, these differences were not significant. The multivariate MR analysis detected no heterogeneity or horizontal pleiotropy.

We also calculated the mediating effect of the gut microbiota in the association of JSW with T2DM and hypertension. A two-step mediation MR analysis showed a significant indirect effect of the *Eubacterium brachy* genus group (β for indirect effect = −0.0535) on the association between JSW and hypertension (**Figure 3**, Panel C; Table S5 in the **Online Supplementary Document**), accounting for 16.64% of the total effect. We observed no significant mediating effect of the gut microbiota taxa in the association between JSW and T2DM.

## DISCUSSION

This study presents genetic evidence linking JSW to CHD risk, identifying traditional CHD risk factors and gut microbiota composition as potential mediators. We confirmed the causal role of JSW in increasing the risk of CHD. The mediation MR analysis suggested that JSW may affect CHD risk by increasing T2DM and hypertension risk. The gut microbiota, specifically *Eubacterium brachy*, mediates the effect of JSW on hypertension, but not on CHD or T2D.

Population data show that workers with JSW are more likely to develop CHD, as well as obesity, T2DM, and hypertension. The results presented here are generally consistent with those of most previous observational and experimental studies and meta-analyses. However, we found little evidence supporting the effect of JSW on obesity and dyslipidaemia. Daghlas et al. [26] reported on the influence of a higher body mass index on JSW status and presumed that the associations of JSW with cardiometabolic disease in previous epidemiological studies might be biased. Therefore, a possible explanation for the increased levels of obesity observed in workers with JSW is the presence of irregular eating habits, not JSW per se.

To date, the mechanisms underlying the relationship between JSW and CHD remain unclear. Our study demonstrated that the effect of JSW on CHD was mediated by an increased risk of T2DM and hypertension. Epidemiological and experimental studies have revealed that disruption of circadian rhythm by JSW results in a series of pathophysiological changes and an increased risk of T2DM and hypertension. Consistent with this, lighting-induced circadian disruption has been shown to result in insulin resistance and glucose intolerance in mice [27]. Additionally, increased levels of inflammatory markers have been reported in workers with JSW [28,29], suggesting that JSW activates the immune and inflammatory systems. Increased psychosocial stress in JSW workers may also lead to abnormal levels of catecholamines, cortisol, leptin, and GLP-1, as well as behavioural changes such as changes in dietary patterns, which may contribute to the development of T2DM and hypertension [30,31].

The gut microbiota consists of approximately 10^13^–10^14^ microorganisms residing in the intestinal tract, 98% of which are bacteria [32]. Dysbiosis of the gut microbiota is linked to nearly 95% of all health conditions, particularly to cardiometabolic diseases [33]. Recent studies concerning the ‘circadian clock-gut microbiota axis’ have proposed a bidirectional regulation between the gut microbiota and circadian rhythm in JSW workers [34,35]. Dysregulation of gut microbiota has also been implicated in the development of CHD and its risk factors [36,37]. In line with these findings, our study suggests that the effect of JSW on hypertension is mediated by abnormal gut microbiota, particularly the abundance of *E. brachy* genus group. Secondary metabolites produced by Actinobacteria species can effectively inhibit the activity of angiotensin-I-converting enzymes and have been proposed as targets for hypertension treatment [38]. *Ruminococcus*2, a genus whose abundance was affected by JSW in our study, has been causally associated with gestational hypertension in another MR study [39]. These results support the effect of the gut microbiota on the increased risk of hypertension caused by JSW, suggesting that targeting the gut microbiota may prevent hypertension and CHD development in workers with JSW.

Our study had several limitations. First, our MR analysis was based on summary-level rather than individual-level data. As such, we cannot distinguish differences in the effects of different types of JSW, such as different times or frequencies of shift work, on CHD risk. Moreover, using the data available, we cannot elucidate in detail the relationships between body mass index or other metabolic factors and JSW or generate a stratified model. Second, information on JSW in the UK biobank cohort was based on self-reported data. We also must acknowledge the limitations of self-reported data, such as potential misclassification and recall bias. More precise and detailed information on work schedules should be collected and assessed in future studies. Third, we could only obtain data at the genus level from the MiBioGen database. A more precise study should be performed in the future to explore the mediating effect of the gut microbiota on the association between JSW and CHD or T2DM. Moreover, dietary patterns are important factors influencing the gut microbiota. Based on prior studies, JSW is known to affect dietary patterns, possibly by increasing psychosocial stress [40]. However, we could not obtain and therefore include data about the participants’ dietary patterns in this study. It remains to be clarified whether the mediating effect of the gut microbiota on the risk of hypertension occurs via the influence of JSW on dietary patterns.

## CONCLUSIONS

Our MR study showed a causal effect of JSW on CHD, with mediation occurring through diabetes, hypertension, and gut microbiota. Our findings therefore support interventions targeting gut microbiota to mitigate hypertension and CHD risk under JSW.

## Additional material


Online Supplementary Document
