# Erratum: Zhang Y, Zhao L, Jia Y, Zhang X, Han Y, Lu P, Yuan H. Mediation Mendelian randomisation study on the effects of shift work on coronary heart disease and traditional risk factors via gut microbiota. J Glob Health. 2024 May 28;14:04110.

**DOI:** 10.7189/jogh.14.1404110err1

**Published:** 2024-12-06

**Authors:** Journal of Global Health Editorial Team



Following the publication of the manuscript ‘Mediation Mendelian randomisation study on the effects of shift work on coronary heart disease and traditional risk factors via gut microbiota’ on 28 May 2024, the authors noted that the asterisk indicating joint first authorship with the first author (Yun Zhang) was omitted for the second author (Lingyun Zhao). This error occurred during the production process due to oversight from both the authors and the technical editors.

The editorial team regrets this error and apologises for any inconvenience it may have caused.

These corrections have been made to the online and the PDF version of the manuscript on 6 December 2024, following the authors’ notice on 23 November 2024.

