# Supplementary material for: Mediation Mendelian randomisation study on the effects of shift work on coronary heart disease and traditional risk factors via gut microbiota
Source: J Glob Health. 2024 May 28;14:04110. doi: 10.7189/jogh.14.04110 (PMC11130565; doi:10.7189/jogh.14.04110)

# Supplementary Documents

## Supplementary Tables

**Page 2** Table S1. STROBE-MR checklist of the present study.

**Page 8** Table S2. Detailed information of genome-wide association studies included in this study.

**Page 9** Table S3. Information about the selected SNPs associated with JSW.

**Page 11** Table S4. MR-estimated effect of JSW on CHD and risk factors.

**Page 13** Table S5. Multivariable and mediation MR analyses of the causal effect of JSW on CHD mediated by traditional risk factors and gut microbiota.

**Page 20** Table S6. MR-estimated effects of JSW on each gut microbiota taxon.

**Page 31** Table S7. Sensitivity analysis of the MR analysis of the causal effect of JSW on gut microbiota.

## Supplementary Figures

**Page 32** Figure S1. Scatter plots and leave-one-out stability test results of the causal estimates of exposure (JSW) on outcomes (coronary heart disease, diabetes, and hypertension).

**Page 33** Figure S2. Scatter plots and leave-one-out stability test results for the causal estimates of exposure (JSW) on gut microbiota.

**Table S1.** STROBE-MR checklist of the present study.

| No. | Section | Checklist item | Position |
|---|---|---|---|
| 1 | Title and abstract | Indicate MR as the study's design in the title and the abstract as a main purpose of the study | Title, Abstract |
| 2 | Background | Explain the scientific background and rationale for the reported study. Explain the exposure and a plausible potential causal relationship between exposure and outcome. Justify why MR is a helpful method to address the study question. | Introduction: paragraph 1-2 |
| 3 | Objectives | State specific objectives clearly, including prespecified causal hypotheses. State that MR is a method that intends to estimate causal effects. | Introduction: paragraph 3 |
| 4a | Study design and data sourses | Setting: Describe the study design (two-sample MR) and the underlying population. Describe the setting, locations, and relevant dates, including periods of recruitment, exposure, follow-up, and data collection. | Materials and methods: *Study design, Data sources*, Figure 1 |
| 4b | | Participants: Report the eligibility criteria and the sources and methods of selection of participants. Report the sample size and whether any power or sample size calculations were carried out prior to the main analysis. | Materials and methods: *Study design, Data sources, Ethical approval* |
| 4c | | Describe measurement, quality control, and selection of genetic variants. | Materials and methods: *Selection of eligible instrumental variables* |

| | | | |
|---|---|---|---|
| 4d | | For each exposure, outcome, and other relevant variables, describe methods of assessment and diagnostic criteria for diseases. | Materials and methods: *Data sources* |
| 4e | | Provide details of ethics committee approval and participant informed consent, if relevant. | Materials and methods: *Ethical approval* |
| 5 | Assumptions | Explicitly state the 3 core instrumental variable (IV) assumptions for the main analysis (relevance, independence, and exclusion restriction), as well assumptions for any additional or sensitivity analysis. | Materials and methods: *Selection of eligible instrumental variables, Statistical methods and sensitivity analysis* |
| 6a | Statistical methods: | Describe how quantitative variables were handled in the analyses. | Materials and methods: *Selection of eligible instrumental variable* |
| 6b | | Describe how genetic variants were handled in the analyses and, if applicable, how their weights were selected | |
| 6c | | Describe the MR estimator and related statistics. Detail the included covariates and, in case of 2-sample MR, whether the same covariate set was used for adjustment in the 2 samples. | Materials and methods: *Statistical methods and sensitivity analysis* |
| 6d | | Explain how missing data were addressed. | |
| 6e | | Indicate how multiple testing was addressed (Bonferroni correction) | |
| 7 | Assessment of assumptions | Describe any methods or prior knowledge used to assess the assumptions or justify their validity | |

| | | | |
|---|---|---|---|
| 8 | Sensitivity analyses and additional analyses | Describe any sensitivity analyses or additional analyses performed (eg, comparison of effect estimates from different approaches, independent replication, bias analytic techniques, validation of instruments, simulations). | |
| 9a | Software and preregistration | Name statistical software and package(s), including version and settings used. | |
| 9b | | State whether the study protocol and details were preregistered (as well as when and where). | N/A |
| 10a | Descriptive data | Report the numbers of individuals at each stage of included studies and reasons for exclusion. Use of a flow diagram. | Figure 1 |
| 10b | | Report summary statistics for phenotypic exposure , outcomes, and other relevant variables (eg, means, SDs, proportions). | Materials and methods: *Study design, Data sources*, Figure 1 |
| 10c | | If the data sources include meta-analyses of previous studies, provide the assessments of heterogeneity across these studies. | N/A |
| 10d | | For 2-sample MR: | |
| | | i. Provide justification of the similarity of the genetic variant–exposure associations between the exposure and outcome samples. | Materials and methods: *Selection of eligible instrumental variables* |
| | | ii. Provide information on the number of individuals who overlap between the exposure and outcome studies. | N/A |

| | | | |
|---|---|---|---|
| 11a | Main results | Report the associations between genetic variant and exposure and between genetic variant and outcome, preferably on an interpretable scale. | Results, Table S3 |
| 11b | | Report MR estimates of the relationship between exposure and outcome and the measures of uncertainty from the MR analysis, on an interpretable scale, such as odds ratio or relative risk per SD difference. | Results-Figure 2-3, Table S4, Table S6 |
| 11c | | If relevant, consider translating estimates of relative risk into absolute risk for a meaningful time period. | N/A |
| 11d | | Consider plots to visualize results (eg, forest plot, scatterplot of associations between genetic variants and outcome vs between genetic variants and exposure). | Figure 2-3, Figure S1-2 |
| 12a | Assessment of assumptions | Report the assessment of the validity of the assumptions by removing confounders-related SNPs. | Results: *Sensitivity analysis* |
| 12b | | Report any additional statistics (eg, assessments of heterogeneity across genetic variants, such as $I^2$, Q statistic). | Results: *Sensitivity analysis* |
| 13a | Sensitivity analyses and additional analyses | Report any sensitivity analyses to assess the robustness of the main results to violations of the assumptions. | Results: *Sensitivity analysis* |
| 13b | | Report results from other sensitivity analyses or additional analyses | Results: *Sensitivity analysis* |

| | | | |
|---|---|---|---|
| 13c | | Report any assessment of the direction of the causal relationship. | Figure 2-3 |
| 13d | | When relevant, report and compare with estimates from other RCTs and meta-analyses. | N/A |
| 13e | | Consider additional plots to visualize results. | All Figure and Table |
| 14 | Key results | Summarize key results with reference to study objectives. | Discussion: Paragraph 1 |
| 15 | Limitations | Discuss limitations of the study, taking into account the validity of the IV assumptions, other sources of potential bias, and imprecision. Discuss both direction and magnitude of any potential bias and any efforts to address them. | Discussion: Paragraph 5 |
| 16a | Interpretation | Meaning: Give a cautious overall interpretation of results in the context of their limitations and in comparison with other studies. | Discussion: Paragraph 1-2 |
| 16b | | Mechanism: Discuss underlying biological mechanisms that could drive a potential causal relationship between the investigated exposure and the outcome, and whether the gene-environment equivalence assumption is reasonable. Use causal language carefully, clarifying that IV estimates may provide causal effects only under certain assumptions. | Discussion |
| 16c | | Clinical relevance: Discuss whether the results have clinical or public policy relevance, and to what extent they inform effect sizes of possible interventions. | |

| | | | |
|---|---|---|---|
| 17 | | Discuss the generalizability of the study results (a) to other populations, (b) across other exposure periods/timings, and (c) across other levels of exposure. | |
| 18 | Funding | Describe sources of funding and the role of funders in the present study. | Acknowledgment: *Funding* |
| 19 | Data and data sharing | Provide the data used to perform all analyses or report where and how the data can be accessed, and reference these sources in the article. | Materials and methods: *Data sources* |
| 20 | Conflicts of interest | All authors should declare all potential conflicts of interest. | Conflicts of Interest |

**Three fundamental assumptions**

a. The instrument variables (IVs) must be associated with exposures;

b. The IVs must not be associated with any confounders, such as age, sex, lifestyle;

c. The IVs must influence the outcomes only through exposures and not through any direct or alternative pathways.

**Table S2.** Detailed information of genome-wide association studies included in this study.

| Year | Dataset | Trait | Abbreviation | Population | Sample size | Numbers of SNPs | PMID | Author | Consortium |
|---|---|---|---|---|---|---|---|---|---|
| 2018 | ukb-b-1712 | Job invovles shift work | JSW | European | 263,315 | 9,851,867 | | Ben Elsworth | MRC-IEU |
| 2015 | ieu-a-7 | Coronary heart disease | CHD | Mixed | 184,305 | 9,455,779 | 26343387 | Nikpay | CARDIoGRAMplusC4D |
| 2021 | finn-b-E4_DM2 | Type 2 diabetes | T2DM | European | 215,654 | 16,380,440 | | | |
| 2021 | finn-b-E4_OBESITY | Obesity | | European | 218,735 | 16,380,465 | | | |
| 2021 | finn-b-I9_HYPTENS | Hypertension | | European | 218,754 | 16,380,466 | | | |
| 2013 | JFx8ih | Total cholesterol | | Mixed | 188,577 | | 24097068 | Willer | |
| 2013 | Z5Cb3y | Low-density lipoprotein cholesterol | LDL | Mixed | 188,577 | | 24097068 | Willer | |
| 2013 | hherZu | Triglyceride | | Mixed | 188,577 | | 24097068 | Willer | |
| 2013 | z4b4uv | High-density lipoprotein cholesterol | HDL | Mixed | 188,577 | | 24097068 | Willer | |
| 2021 | MiBioGen | Gut microbiota | GM | European, Hispanic, Middle Eastern,Asian and African | 18,340 | | 33462485 | Kurilshikov | |

**Table S3.** Information about the selected SNPs associated with JSW.

| SNP | chr | pos | effect_allele | other_allele | eaf | samplesize.exposure | beta.exposure | se.exposure | pval.exposure | R2 | F |
|---|---|---|---|---|---|---|---|---|---|---|---|
| rs666923 | 1 | 234812041 | C | A | 0.207135 | 263315 | 0.0141163 | 0.00283646 | 6.50E-07 | 9.40529E-05 | 24.76766889 |
| rs12129219 | 1 | 247622621 | C | T | 0.207537 | 263315 | 0.0128194 | 0.00287978 | 8.50E-06 | 7.52503E-05 | 19.81588516 |
| rs62172878 | 2 | 154602637 | C | T | 0.076895 | 263315 | 0.019206 | 0.00429974 | 7.90E-06 | 7.57672E-05 | 19.95198732 |
| rs4664365 | 2 | 161600498 | C | T | 0.518708 | 263315 | -0.0102738 | 0.00230906 | 8.60E-06 | 7.51767E-05 | 19.79650297 |
| rs13009008 | 2 | 174043233 | G | A | 0.671752 | 263315 | -0.0119654 | 0.00243581 | 9.00E-07 | 9.16331E-05 | 24.13038917 |
| rs10932655 | 2 | 217209739 | C | A | 0.450518 | 263315 | -0.0107063 | 0.00230369 | 3.40E-06 | 8.202E-05 | 21.59869164 |
| rs10865397 | 2 | 73358292 | G | A | 0.523443 | 263315 | -0.011909 | 0.00233352 | 3.30E-07 | 9.89029E-05 | 26.04499251 |
| rs73092583 | 3 | 71837464 | T | C | 0.419498 | 263315 | -0.0106678 | 0.00236436 | 6.40E-06 | 7.7306E-05 | 20.35725536 |
| rs11922926 | 3 | 23656565 | A | G | 0.004871 | 263315 | 0.0868543 | 0.0189282 | 4.50E-06 | 7.99565E-05 | 21.0552653 |
| rs75814777 | 3 | 171067183 | C | G | 0.028444 | 263315 | -0.0323784 | 0.00688637 | 2.60E-06 | 8.39495E-05 | 22.10684482 |
| rs2087035 | 3 | 131264657 | C | T | 0.169258 | 263315 | -0.0159674 | 0.00306423 | 1.90E-07 | 0.000103111 | 27.15328581 |
| rs1375563 | 3 | 85657946 | T | C | 0.885203 | 263315 | -0.0172282 | 0.00362126 | 2.00E-06 | 8.59503E-05 | 22.63378029 |
| rs34038856 | 3 | 52263967 | A | G | 0.049096 | 263315 | -0.02352 | 0.00530601 | 9.30E-06 | 7.46157E-05 | 19.64876271 |
| rs113510893 | 4 | 94636518 | C | T | 0.068784 | 263315 | 0.0207489 | 0.00462343 | 7.20E-06 | 7.64809E-05 | 20.13994514 |
| rs41534644 | 5 | 121800881 | C | T | 0.146049 | 263315 | -0.0153986 | 0.003246 | 2.10E-06 | 8.5458E-05 | 22.50412574 |
| rs13159978 | 5 | 80547883 | A | G | 0.083399 | 263315 | 0.0187682 | 0.00414676 | 6.00E-06 | 7.7789E-05 | 20.48444235 |
| rs152603 | 5 | 106774922 | G | A | 0.366346 | 263315 | -0.0113181 | 0.0023761 | 1.90E-06 | 8.61598E-05 | 22.68894625 |
| rs115496811 | 5 | 175952776 | T | C | 0.039503 | 263315 | -0.0275965 | 0.00607468 | 5.50E-06 | 7.83703E-05 | 20.63753871 |
| rs76713680 | 6 | 105878480 | T | C | 0.034675 | 263315 | 0.0320549 | 0.00635982 | 4.60E-07 | 9.64677E-05 | 25.40363744 |
| rs1087294 | 6 | 24203724 | G | T | 0.702746 | 263315 | -0.0114844 | 0.00251637 | 5.00E-06 | 7.90966E-05 | 20.82880358 |
| rs9380970 | 6 | 40590738 | A | C | 0.156953 | 263315 | 0.0140895 | 0.00314703 | 7.60E-06 | 7.61168E-05 | 20.04407865 |
| rs9375656 | 6 | 130010372 | C | T | 0.746128 | 263315 | -0.0118034 | 0.00263883 | 7.70E-06 | 7.59771E-05 | 20.00728348 |
| rs10282168 | 7 | 39437185 | A | G | 0.351957 | 263315 | -0.0110603 | 0.00240291 | 4.20E-06 | 8.04541E-05 | 21.18631893 |
| rs1860826 | 7 | 2112506 | A | G | 0.358721 | 263315 | -0.0119023 | 0.00240053 | 7.10E-07 | 9.33537E-05 | 24.5835281 |

| | | | | | | | | | | | |
|---|---|---|---|---|---|---|---|---|---|---|---|
| rs80346402 | 8 | 2584778 | A | G | 0.018327 | 263315 | -0.0394924 | 0.00890063 | 9.10E-06 | 7.47614E-05 | 19.68712325 |
| rs10739536 | 9 | 122151550 | T | A | 0.698464 | 263315 | -0.0111603 | 0.00251445 | 9.10E-06 | 7.48097E-05 | 19.69982786 |
| rs2123202 | 9 | 28153553 | C | A | 0.26047 | 263315 | -0.0118602 | 0.00262893 | 6.40E-06 | 7.72888E-05 | 20.3527305 |
| rs2474711 | 10 | 33441017 | C | G | 0.679375 | 263315 | 0.0117616 | 0.0024578 | 1.70E-06 | 8.69614E-05 | 22.90005009 |
| rs77215157 | 10 | 133599654 | A | G | 0.043115 | 263315 | 0.0268539 | 0.00583403 | 4.20E-06 | 8.04576E-05 | 21.18722701 |
| rs61844343 | 10 | 54557687 | G | T | 0.271581 | 263315 | 0.0126239 | 0.00259585 | 1.20E-06 | 8.98076E-05 | 23.6496439 |
| rs34057425 | 11 | 123341589 | A | T | 0.32332 | 263315 | 0.0115364 | 0.00251462 | 4.50E-06 | 7.99255E-05 | 21.0471154 |
| rs1729200 | 11 | 79986182 | G | A | 0.204588 | 263315 | 0.0130309 | 0.00284574 | 4.70E-06 | 7.96248E-05 | 20.96790733 |
| rs11063070 | 12 | 4377716 | T | C | 0.123618 | 263315 | -0.0162818 | 0.00347529 | 2.80E-06 | 8.3351E-05 | 21.94923718 |
| rs12811792 | 12 | 38054256 | T | A | 0.168576 | 263315 | -0.0144489 | 0.0031307 | 3.90E-06 | 8.08865E-05 | 21.30018451 |
| rs4415916 | 13 | 114261351 | T | G | 0.020808 | 263315 | -0.0386079 | 0.00826928 | 3.00E-06 | 8.27762E-05 | 21.7978503 |
| rs9575634 | 13 | 85238702 | T | C | 0.141606 | 263315 | 0.0153421 | 0.00329452 | 3.20E-06 | 8.2352E-05 | 21.6861283 |
| rs150774726 | 14 | 88402313 | G | T | 0.008599 | 263315 | 0.0588643 | 0.0128172 | 4.40E-06 | 8.00953E-05 | 21.09183411 |
| rs55665482 | 15 | 78066376 | T | C | 0.048328 | 263315 | -0.0251651 | 0.00539579 | 3.10E-06 | 8.25992E-05 | 21.7512362 |
| rs28613960 | 16 | 26213558 | A | G | 0.454628 | 263315 | -0.0114863 | 0.00230665 | 6.40E-07 | 9.4163E-05 | 24.79668402 |
| rs62090078 | 17 | 74006655 | A | G | 0.03446 | 263315 | 0.0282575 | 0.00630101 | 7.30E-06 | 7.63727E-05 | 20.11147096 |
| rs950608 | 17 | 55329305 | A | G | 0.251751 | 263315 | -0.0121723 | 0.00265425 | 4.50E-06 | 7.98641E-05 | 21.03092353 |
| rs57885255 | 19 | 768333 | C | T | 0.018709 | 263315 | 0.0419422 | 0.0090367 | 3.50E-06 | 8.18034E-05 | 21.54167083 |
| rs6039504 | 20 | 9547230 | T | C | 0.163981 | 263315 | -0.0161786 | 0.00308805 | 1.60E-07 | 0.00010423 | 27.44795098 |

**Table S4.** MR-estimated effect of JSW on CHD and risk factors.

| Exposure | Outcome | nSNP | Method | beta | SE | OR (95%CI) | p | p- | Horizontal pleiotropy (MR-Egger) | | | Heterogeneity （Cochran's Q test) | | | | MR-PRESSO |
|---|---|---|---|---|---|---|---|---|---|---|---|---|---|---|---|---|
| | | | | | | | | FDR | Egger intercept | SE | p | Q_MR_Egger | p | Q_IVW | p | p |
| JSW | CHD | 40 | Inverse variance weighted | 0.3078 | 0.1482 | 1.36 (95%CI 1.02-1.82) | 0.04 | | -0.0030 | 0.0069 | 0.66 | 25.6825 | 0.94 | 25.8777 | 0.95 | 0.94 |
| | | | Weighted median | 0.2577 | 0.2031 | 1.29 (95%CI 0.87-1.93) | 0.20 | | | | | | | | | |
| | | | Simple mode | 0.1613 | 0.4298 | 1.18 (95%CI 0.51-2.73) | 0.71 | | | | | | | | | |
| CHD | JSW | 39 | Inverse variance weighted | -0.0049 | 0.0054 | 0.99 (95%CI 0.98-1.01) | 0.37 | | | | | -0.0011 | 0.00 | 0.3732 | 0.11 | 0.12 |
| | | | Weighted median | -0.0002 | 0.0072 | 0.99 (95%CI 0.98-1.01) | 0.98 | | | | | | | | | |
| | | | Simple mode | 0.0058 | 0.0134 | 1.00 (95%CI 0.97-1.03) | 0.67 | | | | | | | | | |
| JSW | T2DM | 42 | Inverse variance weighted | 0.4614 | 0.1701 | 1.59 (95%CI 1.14-2.21) | 0.01 | 0.03 | -0.0032 | 0.0070 | 0.65 | 53.7765 | 0.07 | 54.0610 | 0.08 | 0.10 |
| | | | Weighted median | 0.3926 | 0.2239 | 1.48 (95%CI 0.95-2.30) | 0.08 | 0.28 | | | | | | | | |
| | | | Simple mode | 0.2228 | 0.4549 | 1.25 (95%CI 0.51-3.05) | 0.63 | 0.66 | | | | | | | | |
| JSW | Obesity | 41 | Inverse variance weighted | 0.3911 | 0.2731 | 1.48 (95%CI 0.87-2.53) | 0.15 | 0.25 | -0.0058 | 0.0111 | 0.60 | 51.2125 | 0.09 | 51.5741 | 0.10 | 0.10 |
| | | | Weighted median | 0.4283 | 0.3539 | 1.53 (95%CI 0.77-3.07) | 0.23 | 0.32 | | | | | | | | |
| | | | Simple mode | 1.0097 | 0.7737 | 2.74 (95%CI 0.60-12.50) | 0.20 | 0.56 | | | | | | | | |
| JSW | Hypertension | 43 | Inverse variance weighted | 0.3604 | 0.1357 | 1.43 (95%CI 1.10-1.87) | 0.01 | 0.03 | -0.0079 | 0.0055 | 0.16 | 44.5664 | 0.32 | 46.8023 | 0.28 | 0.28 |
| | | | Weighted median | 0.3449 | 0.1932 | 1.41 (95%CI 0.97-2.06) | 0.07 | 0.28 | | | | | | | | |
| | | | Simple mode | 1.0870 | 0.4483 | 2.97 (95%CI 1.23-7.14) | 0.02 | 0.14 | | | | | | | | |
| JSW | TC | 11 | Inverse variance weighted | 0.1943 | 0.1455 | 1.21 (95%CI 0.91-1.62) | 0.18 | 0.25 | 0.0066 | 0.0076 | 0.41 | 4.9540 | 0.84 | 5.6929 | 0.84 | 0.84 |
| | | | Weighted median | 0.2378 | 0.1897 | 1.27 (95%CI 0.87-1.84) | 0.21 | 0.32 | | | | | | | | |
| | | | Simple mode | 0.3720 | 0.2969 | 1.45 (95%CI 0.81-2.60) | 0.24 | 0.56 | | | | | | | | |
| JSW | LDL | 13 | Inverse variance weighted | 0.2501 | 0.1390 | 1.28 (95%CI 0.98-1.69) | 0.07 | 0.17 | 0.0029 | 0.0071 | 0.69 | 9.0434 | 0.62 | 9.2154 | 0.68 | 0.71 |
| | | | Weighted median | 0.2523 | 0.1860 | 1.29 (95%CI 0.89-1.85) | 0.18 | 0.32 | | | | | | | | |
| | | | Simple mode | 0.3095 | 0.3048 | 1.36 (95%CI 0.75-2.48) | 0.33 | 0.58 | | | | | | | | |

| | | | | | | | | | | | | | | | |
|---|---|---|---|---|---|---|---|---|---|---|---|---|---|---|---|
| JSW | TG | 12 | Inverse variance weighted | 0.1509 | 0.1589 | 1.16 (95%CI 0.85-1.59) | 0.34 | 0.34 | 0.0094 | 0.0078 | 0.26 | 14.5298 | 0.15 | 16.6110 | 0.12 | 0.13 |
| | | | Weighted median | 0.1137 | 0.1786 | 1.12 (95%CI 0.79-1.59) | 0.52 | 0.52 | | | | | | | | |
| | | | Simple mode | 0.1447 | 0.3195 | 1.16 (95%CI 0.62-2.16) | 0.66 | 0.66 | | | | | | | | |
| JSW | HDL | 13 | Inverse variance weighted | -0.1360 | 0.1282 | 0.87 (95%CI 0.68-1.12) | 0.29 | 0.34 | 0.0050 | 0.0067 | 0.47 | 10.6535 | 0.47 | 11.2197 | 0.51 | 0.50 |
| | | | Weighted median | -0.1294 | 0.1804 | 0.88 (95%CI 0.62-1.25) | 0.47 | 0.52 | | | | | | | | |
| | | | Simple mode | 0.1920 | 0.3483 | 1.21 (95%CI 0.61-2.40) | 0.59 | 0.66 | | | | | | | | |

CHD: coronary heart disease

JSW: jobs invovling shift work

T2DM: type 2 diabetes

TC: total cholesterol

HDL: high-density lipoprotein cholesterol

LDL: low-density lipoprotein cholesterol

TG: triglyceride

**Table S5.** Multivariable and mediation MR analyses of the causal effect of JSW on CHD mediated by traditional risk factors and gut microbiota.

| outcome | id.exposure | exposure | number of | IVW | | | Heterogeneity test | | MR-Egger | | | Heterogeneity test | | Mediation effect | Mediation effect | Proportion mediated |
|---|---|---|---|---|---|---|---|---|---|---|---|---|---|---|---|---|
| | | | variants | b (95%CI) | se | p | statistic | p | b (95%CI) | se | p | statistic | p | β1 × β2 | 95%CI | β1 × β2/(β1 × β2+β3) |
| CHD | ukb-b-1712 | JSW | 71 | 0.301 [-0.001, 0.603] | 0.154 | 0.05 | 73.9735 | 0.32 | 0.264 [-0.241, 0.769] | 0.257 | 0.31 | 73.9371 | 0.29 | 0.0406 | 0.0090, 0.0830 | 0.118857134 |
| | finn-b-E4_DM2 | T2DM | 71 | 0.088 [0.041, 0.135] | 0.024 | <0.001 | | | 0.086 [0.035, 0.138] | 0.026 | 0.00 | | | | | |
| | | (intercept) | | | | | | | 0.000 [-0.005, 0.006] | 0.003 | 0.86 | | | | | |
| CHD | ukb-b-1712 | JSW | 78 | 0.278 [-0.057, 0.612] | 0.171 | 0.10 | 98.7714 | 0.04 | 0.344 [-0.154, 0.842] | 0.254 | 0.18 | 98.6058 | 0.04 | 0.0483 | 0.0114, 0.0947 | 0.148006581 |
| | finn-b-I9_HYPTENS | Hypertension | 78 | 0.134 [0.076, 0.191] | 0.029 | <0.001 | | | 0.135 [0.077, 0.193] | 0.030 | <0.001 | | | | | |
| | | (intercept) | | | | | | | -0.001 [-0.006, 0.004] | 0.002 | 0.72 | | | | | |
| CHD | ukb-b-1712 | JSW | 104 | 0.312 [-0.048, 0.672] | 0.184 | 0.09 | ####### | 0.00 | 0.353 [-0.154, 0.861] | 0.259 | 0.17 | 143.8480 | 0.00 | | | |
| | finn-b-E4_DM2 | T2DM | 104 | 0.069 [0.010, 0.128] | 0.030 | 0.02 | | | 0.071 [0.009, 0.132] | 0.031 | 0.02 | | | | | |
| | finn-b-I9_HYPTENS | Hypertension | | 0.123 [0.060, 0.185] | 0.032 | <0.001 | | | 0.123 [0.060, 0.186] | 0.032 | <0.001 | | | | | |
| | | (intercept) | | | | | | | 0.000 [-0.005, 0.004] | 0.002 | 0.82 | | | | | |
| CHD | ukb-b-1712 | JSW | 45 | 0.324 [0.005, 0.643] | 0.163 | 0.05 | 40.2734 | 0.59 | 0.228 [-0.398, 0.853] | 0.319 | 0.48 | 40.1498 | 0.55 | -0.0241 | -0.0808, 0.0124 | 0.069336594 |
| | | phylum Actinobacteria | 45 | -0.063 [-0.158, 0.031] | 0.048 | 0.19 | | | -0.064 [-0.158, 0.030] | 0.048 | 0.18 | | | | | |

| | | | | | | | | | | | | | | | | |
|---|---|---|---|---|---|---|---|---|---|---|---|---|---|---|---|---|
| | | (intercept) | | | | | | | 0.001 [-0.005, 0.008] | 0.003 | 0.73 | | | | | |
| CHD | ukb-b-1712 | JSW | 48 | 0.284 [-0.029, 0.597] | 0.160 | 0.08 | 37.3520 | 0.81 | 0.610 [-0.115, 1.334] | 0.370 | 0.10 | 36.3960 | 0.82 | 0.0116 | -0.0252, 0.0581 | 0.039366282 |
| | | class Coriobacteriia | 48 | 0.029 [-0.057, 0.115] | 0.044 | 0.51 | | | 0.023 [-0.064, 0.110] | 0.044 | 0.61 | | | | | |
| | | (intercept) | | | | | | | -0.004 [-0.012, 0.004] | 0.004 | 0.33 | | | | | |
| CHD | ukb-b-1712 | JSW | 48 | 0.284 [-0.029, 0.597] | 0.160 | 0.08 | 37.3520 | 0.81 | 0.610 [-0.115, 1.334] | 0.370 | 0.10 | 36.3960 | 0.82 | 0.0116 | -0.0252, 0.0581 | 0.039366282 |
| | | order Coriobacteriales | 48 | 0.029 [-0.057, 0.115] | 0.044 | 0.51 | | | 0.023 [-0.064, 0.110] | 0.044 | 0.61 | | | | | |
| | | (intercept) | | | | | | | -0.004 [-0.012, 0.004] | 0.004 | 0.33 | | | | | |
| CHD | ukb-b-1712 | JSW | 48 | 0.284 [-0.029, 0.597] | 0.160 | 0.08 | 37.3520 | 0.81 | 0.610 [-0.115, 1.334] | 0.370 | 0.10 | 36.3960 | 0.82 | 0.0116 | -0.0252, 0.0581 | 0.039366282 |
| | | family Coriobacteriaceae | 48 | 0.029 [-0.057, 0.115] | 0.044 | 0.51 | | | 0.023 [-0.064, 0.110] | 0.044 | 0.61 | | | | | |
| | | (intercept) | | | | | | | -0.004 [-0.012, 0.004] | 0.004 | 0.33 | | | | | |
| CHD | ukb-b-1712 | JSW | 41 | 0.213 [-0.110, 0.537] | 0.165 | 0.20 | 33.1470 | 0.73 | 0.837 [0.109, 1.564] | 0.371 | 0.02 | 29.6340 | 0.83 | 0.0262 | -0.0301, 0.0982 | 0.109612937 |
| | | genus *Eubacterium brachy* group | 41 | 0.025 [-0.029, 0.079] | 0.027 | 0.36 | | | 0.027 [-0.026, 0.081] | 0.027 | 0.32 | | | | | |
| | | (intercept) | | | | | | | -0.008 [-0.016, 0.000] | 0.004 | 0.06 | | | | | |
| CHD | ukb-b-1712 | JSW | 44 | 0.234 [-0.082, 0.550] | 0.161 | 0.15 | 29.0741 | 0.93 | 0.775 [0.092, 1.458] | 0.348 | 0.03 | 26.0039 | 0.97 | -0.0229 | -0.0840, 0.0185 | 0.088991755 |

| | | | | | | | | | | | | | | | | |
|---|---|---|---|---|---|---|---|---|---|---|---|---|---|---|---|---|
| | | genus *Ruminococcus gnavus* group | 44 | 0.032 [-0.025, 0.089] | 0.029 | 0.27 | | | 0.035 [-0.022, 0.092] | 0.029 | 0.23 | | | | | |
| | | (intercept) | | | | | | | -0.007 [-0.014, 0.001] | 0.004 | 0.08 | | | | | |
| CHD | ukb-b-1712 | JSW | 41 | 0.289 [-0.031, 0.609] | 0.163 | 0.08 | 30.3255 | 0.84 | 0.158 [-0.595, 0.912] | 0.384 | 0.68 | 30.1835 | 0.81 | -0.0293 | -0.0941, 0.0118 | 0.091939953 |
| | | genus *Lachnoclostridium* | 41 | 0.078 [-0.029, 0.185] | 0.055 | 0.15 | | | 0.073 [-0.038, 0.184] | 0.057 | 0.20 | | | | | |
| | | (intercept) | | | | | | | 0.002 [-0.007, 0.010] | 0.004 | 0.71 | | | | | |
| CHD | ukb-b-1712 | JSW | 44 | 0.257 [-0.060, 0.574] | 0.162 | 0.11 | 32.7855 | 0.85 | 0.690 [-0.051, 1.431] | 0.378 | 0.07 | 31.1786 | 0.87 | -0.0148 | -0.0875, 0.0520 | 0.054488765 |
| | | genus *Roseburia* | 44 | 0.023 [-0.076, 0.122] | 0.051 | 0.65 | | | 0.033 [-0.067, 0.134] | 0.051 | 0.52 | | | | | |
| | | (intercept) | | | | | | | -0.005 [-0.014, 0.003] | 0.004 | 0.21 | | | | | |
| CHD | ukb-b-1712 | JSW | 45 | 0.286 [-0.029, 0.601] | 0.161 | 0.08 | 35.1942 | 0.80 | -0.116 [-0.829, 0.596] | 0.363 | 0.75 | 33.6708 | 0.82 | 0.0299 | -0.0076, 0.0895 | 0.094770633 |
| | | genus *Ruminococcus2* | 45 | 0.067 [-0.015, 0.150] | 0.042 | 0.11 | | | 0.064 [-0.019, 0.146] | 0.042 | 0.13 | | | | | |
| | | (intercept) | | | | | | | 0.005 [-0.003, 0.013] | 0.004 | 0.22 | | | | | |
| T2DM | ukb-b-1712 | JSW | 47 | 0.312 [-0.119, 0.744] | 0.220 | 0.16 | 74.6523 | 0.00 | 0.842 [0.002, 1.682] | 0.429 | 0.05 | 71.3125 | 0.01 | -0.0211 | -0.0908, 0.0299 | 0.063269866 |
| | | phylum Actinobacteria | 47 | -0.055 [-0.182, 0.072] | 0.065 | 0.40 | | | -0.048 [-0.174, 0.078] | 0.064 | 0.46 | | | | | |
| | | (intercept) | | | | | | | -0.007 [-0.016, 0.002] | 0.005 | 0.15 | | | | | |

| | | | | | | | | | | | | | | | | | |
|---|---|---|---|---|---|---|---|---|---|---|---|---|---|---|---|---|---|
| T2DM | ukb-b-1712 | JSW | 48 | 0.311 [-0.101, 0.723] | 0.210 | 0.14 | 71.8031 | 0.01 | -0.091 [-1.097, 0.915] | 0.513 | 0.86 | 70.6450 | 0.01 | 0.0221 | -0.0256, 0.0878 | | 0.066269082 |
| | | class Coriobacteriia | 48 | 0.055 [-0.110, 0.120] | 0.059 | 0.93 | | | 0.014 [-0.103, 0.131] | 0.060 | 0.82 | | | | | | |
| | | (intercept) | | | | | | | 0.005 [-0.006, 0.017] | 0.006 | 0.39 | | | | | | |
| T2DM | ukb-b-1712 | JSW | 48 | 0.311 [-0.101, 0.723] | 0.210 | 0.14 | 71.8031 | 0.01 | -0.091 [-1.097, 0.915] | 0.513 | 0.86 | 70.6450 | 0.01 | 0.0221 | -0.0256, 0.0878 | | 0.066269082 |
| | | order Coriobacteriales | 48 | 0.055 [-0.110, 0.120] | 0.059 | 0.93 | | | 0.014 [-0.103, 0.131] | 0.060 | 0.82 | | | | | | |
| | | (intercept) | | | | | | | 0.005 [-0.006, 0.017] | 0.006 | 0.39 | | | | | | |
| T2DM | ukb-b-1712 | JSW | 48 | 0.311 [-0.101, 0.723] | 0.210 | 0.14 | 71.8031 | 0.01 | -0.091 [-1.097, 0.915] | 0.513 | 0.86 | 70.6450 | 0.01 | 0.0221 | -0.0256, 0.0878 | | 0.066269082 |
| | | family Coriobacteriaceae | 48 | 0.055 [-0.110, 0.120] | 0.059 | 0.93 | | | 0.014 [-0.103, 0.131] | 0.060 | 0.82 | | | | | | |
| | | (intercept) | | | | | | | 0.005 [-0.006, 0.017] | 0.006 | 0.39 | | | | | | |
| T2DM | ukb-b-1712 | JSW | 43 | 0.282 [-0.176, 0.740] | 0.234 | 0.23 | 73.7647 | 0.00 | 0.857 [-0.166, 1.881] | 0.522 | 0.10 | 71.0774 | 0.00 | -0.0241 | -0.1228, 0.0599 | | 0.078804849 |
| | | genus *Eubacterium brachy* group | 43 | -0.023 [-0.099, 0.053] | 0.039 | 0.56 | | | -0.024 [-0.100, 0.052] | 0.039 | 0.53 | | | | | | |
| | | (intercept) | | | | | | | -0.007 [-0.019, 0.004] | 0.006 | 0.22 | | | | | | |
| T2DM | ukb-b-1712 | JSW | 45 | 0.307 [-0.124, 0.738] | 0.220 | 0.16 | 71.5728 | 0.00 | 0.311 [-0.653, 1.274] | 0.492 | 0.53 | 71.5727 | 0.00 | -0.0414 | -0.1335, 0.0169 | | 0.118906346 |
| | | genus *Ruminococcus gnavus* group | 45 | 0.058 [-0.022, 0.138] | 0.041 | 0.15 | | | 0.058 [-0.023, 0.140] | 0.041 | 0.16 | | | | | | |
| | | (intercept) | | | | | | | 0.000 [-0.011, 0.011] | 0.005 | 0.99 | | | | | | |

| | | | | | | | | | | | | | | | | |
|---|---|---|---|---|---|---|---|---|---|---|---|---|---|---|---|---|
| T2DM | ukb-b-1712 | JSW | 42 | 0.331 [-0.087, 0.749] | 0.213 | 0.12 | 60.9646 | 0.02 | -0.064 [-1.085, 0.958] | 0.521 | 0.90 | 59.9056 | 0.02 | -0.0244 | -0.1033, 0.3303 | 0.068613305 |
| | | genus *Lachnoclostridium* | 42 | 0.065 [-0.081, 0.211] | 0.075 | 0.39 | | | 0.044 [-0.110, 0.199] | 0.079 | 0.58 | | | | | |
| | | (intercept) | | | | | | | 0.005 [-0.007, 0.017] | 0.006 | 0.41 | | | | | |
| T2DM | ukb-b-1712 | JSW | 45 | 0.302 [-0.163, 0.767] | 0.237 | 0.20 | 83.4119 | 0.00 | 0.372 [-0.754, 1.497] | 0.574 | 0.52 | 83.3767 | 0.00 | 0.0129 | -0.0882, 0.1191 | 0.04090083 |
| | | genus *Roseburia* | 45 | -0.020 [-0.170, 0.129] | 0.076 | 0.79 | | | -0.019 [-0.171, 0.134] | 0.078 | 0.81 | | | | | |
| | | (intercept) | | | | | | | -0.001 [-0.014, 0.012] | 0.007 | 0.89 | | | | | |
| T2DM | ukb-b-1712 | JSW | 46 | 0.279 [-0.155, 0.713] | 0.222 | 0.21 | 75.8055 | 0.00 | 0.593 [-0.385, 1.571] | 0.499 | 0.24 | 74.9416 | 0.00 | 0.0487 | -0.0046, 0.1337 | 0.148641811 |
| | | genus *Ruminococcus2* | 46 | 0.109 [-0.001, 0.219] | 0.056 | 0.05 | | | 0.116 [0.003, 0.228] | 0.057 | 0.04 | | | | | |
| | | (intercept) | | | | | | | -0.004 [-0.015, 0.007] | 0.006 | 0.48 | | | | | |
| Hypertension | ukb-b-1712 | JSW | 47 | 0.271 [-0.107, 0.649] | 0.193 | 0.16 | 73.9889 | 0.00 | 0.007 [-0.739, 0.754] | 0.381 | 0.98 | 72.9177 | 0.00 | -0.0330 | -0.1030, 0.0109 | 0.092300608 |
| | | phylum Actinobacteria | 47 | -0.086 [-0.197, 0.026] | 0.057 | 0.13 | | | -0.089 [-0.201, -0.023] | 0.057 | 0.12 | | | | | |
| | | (intercept) | | | | | | | 0.003 [-0.005, 0.011] | 0.004 | 0.42 | | | | | |
| Hypertension | ukb-b-1712 | JSW | 48 | 0.262 [-0.168, 0.691] | 0.219 | 0.23 | ####### | <0.001 | -0.600 [-1.620, 0.419] | 0.520 | 0.25 | 93.8299 | <0.001 | 0.0245 | -0.0446, 0.0696 | 0.10054325 |
| | | class Coriobacteriia | 48 | 0.022 [-0.099, 0.142] | 0.061 | 0.73 | | | 0.040 [-0.079, 0.159] | 0.061 | 0.51 | | | | | |
| | | (intercept) | | | | | | | 0.011 [-0.001, 0.022] | 0.006 | 0.07 | | | | | |

| | | | | | | | | | | | | | | | | |
|---|---|---|---|---|---|---|---|---|---|---|---|---|---|---|---|---|
| Hypertension | ukb-b-1712 | JSW | 48 | 0.262 [-0.168, 0.691] | 0.219 | 0.23 | ####### | <0.001 | -0.600 [-1.620, 0.419] | 0.520 | 0.25 | 93.8299 | <0.001 | 0.0245 | -0.0446, 0.0696 | 0.10054325 |
| | | order Coriobacteriales | 48 | 0.022 [-0.099, 0.142] | 0.061 | 0.73 | | | 0.040 [-0.079, 0.159] | 0.061 | 0.51 | | | | | |
| | | (intercept) | | | | | | | 0.011 [-0.001, 0.022] | 0.006 | 0.07 | | | | | |
| Hypertension | ukb-b-1712 | JSW | 48 | 0.262 [-0.168, 0.691] | 0.219 | 0.23 | ####### | <0.001 | -0.600 [-1.620, 0.419] | 0.520 | 0.25 | 93.8299 | <0.001 | 0.0245 | -0.0446, 0.0696 | 0.10054325 |
| | | family Coriobacteriaceae | 48 | 0.022 [-0.099, 0.142] | 0.061 | 0.73 | | | 0.040 [-0.079, 0.159] | 0.061 | 0.51 | | | | | |
| | | (intercept) | | | | | | | 0.011 [-0.001, 0.022] | 0.006 | 0.07 | | | | | |
| Hypertension | ukb-b-1712 | JSW | 43 | 0.268 [-0.033, 0.568] | 0.153 | 0.08 | 54.3254 | 0.08 | 0.742 [-0.027, 1.511] | 0.392 | 0.06 | 51.9542 | 0.10 | -0.0535 | -1.3334e-1, -5.0082e-5 | 0.16638797 |
| | | genus *Eubacterium brachy* group | 43 | -0.051 [-0.101,-0.001] | 0.025 | 0.05 | | | -0.052 [-0.109, 0.005] | 0.029 | 0.08 | | | | | |
| | | (intercept) | | | | | | | -0.006 [-.015, 0.003] | 0.004 | 0.18 | | | | | |
| Hypertension | ukb-b-1712 | JSW | 45 | 0.272 [-0.022, 0.565] | 0.150 | 0.07 | 41.8241 | 0.52 | 0.027 [-0.622, 0.676] | 0.331 | 0.94 | 41.1382 | 0.51 | -0.0279 | -0.0904, 0.0119 | 0.09290553 |
| | | genus *Ruminococcus gnavus* group | 45 | 0.039 [-0.015, 0.094] | 0.028 | 0.16 | | | 0.037 [-0.017, 0.092] | 0.028 | 0.18 | | | | | |
| | | (intercept) | | | | | | | 0.003 [-0.004, 0.010] | 0.004 | 0.41 | | | | | |
| Hypertension | ukb-b-1712 | JSW | 42 | 0.268 [-0.029, 0.566] | 0.152 | 0.08 | 43.9392 | 0.31 | 0.288 [-0.483, 1.059] | 0.393 | 0.46 | 43.9358 | 0.27 | -0.0218 | -0.0802, 0.0180 | 0.075090563 |

| | | | | | | | | | | | | | | | | |
|---|---|---|---|---|---|---|---|---|---|---|---|---|---|---|---|---|
| | | genus *Lachnoclostridium* | 42 | 0.058 [-0.046, 0.163] | 0.053 | 0.28 | | | 0.059 [-0.058, 0.176] | 0.060 | 0.32 | | | | | |
| | | (intercept) | | | | | | | 0.000 [-0.009, 0.009] | 0.005 | 0.96 | | | | | |
| Hypertension | ukb-b-1712 | JSW | 45 | 0.257 [-0.037, 0.551] | 0.150 | 0.09 | 54.5771 | 0.11 | -0.299 [-1.078, 0.480] | 0.397 | 0.45 | 51.6622 | 0.15 | -0.0155 | -0.0843, 0.0472 | 0.056723459 |
| | | genus *Roseburia* | 45 | 0.024 [-0.071, 0.118] | 0.048 | 0.62 | | | 0.011 [-0.095, 0.117] | 0.054 | 0.84 | | | | | |
| | | (intercept) | | | | | | | 0.007 [-0.002, 0.016] | 0.005 | 0.12 | | | | | |
| Hypertension | ukb-b-1712 | JSW | 46 | 0.254 [-0.037, 0.545] | 0.148 | 0.09 | 53.1198 | 0.16 | 0.250 [-0.474, 0.975] | 0.370 | 0.50 | 53.1197 | 0.14 | 0.0089 | -0.0272, 0.0526 | 0.033992504 |
| | | genus *Ruminococcus2* | 46 | 0.020 [-0.054, 0.094] | 0.038 | 0.60 | | | 0.020 [-0.064, 0.103] | 0.043 | 0.65 | | | | | |
| | | (intercept) | | | | | | | 0.000 [-0.008, 0.008] | 0.004 | 0.99 | | | | | |

CHD: coronary heart disease

JSW: jobs invovling shift work

T2DM: type 2 diabetes

**Table S6.** MR-estimated effects of JSW on each gut microbiota taxon.

| Exposure | Outcome | Nsnp | Inverse variance weighted | | | | | | | Weighted_median | | | | | | | Simple_mode | | | | | | |
|---|---|---|---|---|---|---|---|---|---|---|---|---|---|---|---|---|---|---|---|---|---|---|---|
| | | | beta | SE | p | OR | CI_low | CI_high | p-FDR | beta | SE | p | OR | CI_low | CI_high | p-FDR | beta | SE | p | OR | CI_low | CI_high | p-FDR |
| JSW | phylum Actinobacteria id 400 | 28 | 0.38 | 0.19 | 0.05 | 1.47 | 1.01 | 2.14 | 0.32 | 0.23 | 0.26 | 0.39 | 1.26 | 0.75 | 2.10 | 0.70 | 0.14 | 0.60 | 0.82 | 1.15 | 0.35 | 3.74 | 0.92 |
| JSW | phylum Bacteroidetes id 905 | 28 | -0.11 | 0.19 | 0.56 | 0.90 | 0.62 | 1.29 | 0.61 | -0.03 | 0.26 | 0.92 | 0.97 | 0.58 | 1.63 | 0.94 | 0.30 | 0.60 | 0.63 | 1.34 | 0.41 | 4.35 | 0.92 |
| JSW | phylum Cyanobacteria id 1500 | 28 | 0.33 | 0.31 | 0.29 | 1.39 | 0.76 | 2.54 | 0.55 | 0.53 | 0.43 | 0.22 | 1.70 | 0.73 | 3.99 | 0.70 | 0.49 | 0.83 | 0.56 | 1.63 | 0.32 | 8.33 | 0.92 |
| JSW | phylum Euryarchaeota id 55 | 26 | 0.42 | 0.47 | 0.37 | 1.52 | 0.61 | 3.81 | 0.55 | 0.05 | 0.60 | 0.94 | 1.05 | 0.32 | 3.40 | 0.94 | 0.99 | 1.28 | 0.45 | 2.69 | 0.22 | 33.01 | 0.92 |
| JSW | phylum Firmicutes id 1672 | 28 | -0.10 | 0.19 | 0.61 | 0.91 | 0.63 | 1.31 | 0.61 | -0.03 | 0.26 | 0.91 | 0.97 | 0.59 | 1.61 | 0.94 | 0.05 | 0.55 | 0.92 | 1.06 | 0.36 | 3.08 | 0.92 |
| JSW | phylum Lentisphaerae id 2238 | 27 | -0.34 | 0.37 | 0.36 | 0.71 | 0.35 | 1.47 | 0.55 | -0.72 | 0.50 | 0.15 | 0.49 | 0.18 | 1.29 | 0.70 | -0.93 | 1.07 | 0.39 | 0.40 | 0.05 | 3.20 | 0.92 |
| JSW | phylum Proteobacteria id 2375 | 28 | -0.34 | 0.19 | 0.07 | 0.72 | 0.50 | 1.03 | 0.32 | -0.08 | 0.24 | 0.75 | 0.93 | 0.57 | 1.49 | 0.94 | 0.07 | 0.52 | 0.89 | 1.07 | 0.39 | 2.94 | 0.92 |
| JSW | phylum Tenericutes id 3919 | 28 | 0.18 | 0.26 | 0.48 | 1.20 | 0.72 | 2.00 | 0.61 | 0.34 | 0.35 | 0.32 | 1.41 | 0.71 | 2.78 | 0.70 | 0.51 | 0.70 | 0.48 | 1.66 | 0.42 | 6.58 | 0.92 |
| JSW | phylum Verrucomicrobia id 3982 | 28 | -0.28 | 0.22 | 0.22 | 0.76 | 0.49 | 1.18 | 0.55 | -0.31 | 0.32 | 0.34 | 0.74 | 0.39 | 1.37 | 0.70 | 0.21 | 0.65 | 0.75 | 1.23 | 0.35 | 4.40 | 0.92 |
| JSW | class Actinobacteria id 419 | 28 | 0.09 | 0.23 | 0.71 | 1.09 | 0.69 | 1.71 | 0.94 | -0.16 | 0.28 | 0.57 | 0.85 | 0.49 | 1.48 | 0.83 | -0.77 | 0.60 | 0.21 | 0.46 | 0.14 | 1.51 | 0.69 |
| JSW | class Alphaproteobacteria id 2379 | 28 | -0.02 | 0.28 | 0.94 | 0.98 | 0.57 | 1.68 | 0.99 | -0.01 | 0.37 | 0.98 | 0.99 | 0.48 | 2.05 | 0.98 | -0.20 | 0.71 | 0.79 | 0.82 | 0.20 | 3.33 | 0.84 |
| JSW | class Bacilli id 1673 | 28 | 0.00 | 0.19 | 0.99 | 1.00 | 0.69 | 1.46 | 0.99 | 0.06 | 0.25 | 0.82 | 1.06 | 0.65 | 1.74 | 0.98 | 0.29 | 0.49 | 0.56 | 1.34 | 0.51 | 3.51 | 0.69 |
| JSW | class Bacteroidia id 912 | 28 | -0.11 | 0.19 | 0.57 | 0.90 | 0.63 | 1.29 | 0.83 | 0.02 | 0.26 | 0.93 | 1.02 | 0.61 | 1.72 | 0.98 | 0.34 | 0.56 | 0.55 | 1.40 | 0.47 | 4.21 | 0.69 |
| JSW | class Betaproteobacteria id 2867 | 28 | -0.22 | 0.19 | 0.25 | 0.80 | 0.55 | 1.17 | 0.69 | -0.28 | 0.26 | 0.28 | 0.76 | 0.45 | 1.26 | 0.59 | -0.54 | 0.51 | 0.30 | 0.58 | 0.22 | 1.58 | 0.69 |
| JSW | class Clostridia id 1859 | 28 | 0.03 | 0.19 | 0.88 | 1.03 | 0.72 | 1.48 | 0.99 | 0.25 | 0.26 | 0.33 | 1.28 | 0.78 | 2.12 | 0.59 | 0.45 | 0.53 | 0.40 | 1.57 | 0.55 | 4.47 | 0.69 |
| JSW | class Coriobacteriia id 809 | 28 | 0.40 | 0.19 | 0.03 | 1.49 | 1.03 | 2.17 | 0.55 | 0.36 | 0.27 | 0.18 | 1.44 | 0.84 | 2.45 | 0.59 | 0.40 | 0.59 | 0.50 | 1.49 | 0.47 | 4.72 | 0.69 |
| JSW | class Deltaproteobacteria id 3087 | 28 | -0.19 | 0.22 | 0.39 | 0.83 | 0.54 | 1.27 | 0.69 | -0.37 | 0.29 | 0.21 | 0.69 | 0.39 | 1.22 | 0.59 | -0.56 | 0.59 | 0.34 | 0.57 | 0.18 | 1.79 | 0.69 |
| JSW | class Erysipelotrichia id 2147 | 28 | -0.22 | 0.21 | 0.30 | 0.80 | 0.53 | 1.22 | 0.69 | -0.29 | 0.26 | 0.27 | 0.75 | 0.45 | 1.25 | 0.59 | -0.36 | 0.48 | 0.46 | 0.70 | 0.27 | 1.80 | 0.69 |
| JSW | class Gammaproteobacteria id 3303 | 28 | -0.33 | 0.20 | 0.10 | 0.72 | 0.49 | 1.06 | 0.69 | -0.20 | 0.27 | 0.45 | 0.82 | 0.49 | 1.38 | 0.72 | -0.41 | 0.55 | 0.46 | 0.66 | 0.23 | 1.93 | 0.69 |
| JSW | class Lentisphaeria id 2250 | 27 | -0.34 | 0.37 | 0.35 | 0.71 | 0.34 | 1.46 | 0.69 | -0.70 | 0.50 | 0.16 | 0.49 | 0.18 | 1.33 | 0.59 | -1.02 | 1.08 | 0.35 | 0.36 | 0.04 | 2.99 | 0.69 |
| JSW | class Melainabacteria id 1589 | 28 | 0.46 | 0.38 | 0.22 | 1.59 | 0.76 | 3.33 | 0.69 | 1.03 | 0.49 | 0.03 | 2.80 | 1.08 | 7.24 | 0.55 | 1.33 | 0.97 | 0.18 | 3.77 | 0.57 | 25.01 | 0.69 |
| JSW | class Methanobacteria id 119 | 26 | 0.38 | 0.44 | 0.39 | 1.46 | 0.62 | 3.45 | 0.69 | 0.19 | 0.60 | 0.75 | 1.21 | 0.38 | 3.90 | 0.98 | 0.61 | 1.21 | 0.62 | 1.83 | 0.17 | 19.73 | 0.71 |
| JSW | class Mollicutes id 3920 | 28 | 0.18 | 0.26 | 0.48 | 1.20 | 0.72 | 2.00 | 0.77 | 0.34 | 0.35 | 0.33 | 1.41 | 0.70 | 2.82 | 0.59 | 0.51 | 0.74 | 0.50 | 1.66 | 0.39 | 7.10 | 0.69 |

| | | | | | | | | | | | | | | | | | | | | | | | |
|---|---|---|---|---|---|---|---|---|---|---|---|---|---|---|---|---|---|---|---|---|---|---|---|
| JSW | class Negativicutes id 2164 | 28 | 0.00 | 0.19 | 0.99 | 1.00 | 0.70 | 1.44 | 0.99 | -0.03 | 0.26 | 0.90 | 0.97 | 0.58 | 1.61 | 0.98 | -0.10 | 0.54 | 0.86 | 0.91 | 0.31 | 2.63 | 0.86 |
| JSW | class Verrucomicrobiae id 4029 | 28 | -0.33 | 0.23 | 0.14 | 0.72 | 0.46 | 1.12 | 0.69 | -0.32 | 0.31 | 0.30 | 0.72 | 0.39 | 1.34 | 0.59 | -1.00 | 0.63 | 0.12 | 0.37 | 0.11 | 1.26 | 0.69 |
| JSW | order Actinomycetales id 420 | 28 | 0.17 | 0.28 | 0.55 | 1.18 | 0.68 | 2.06 | 0.81 | 0.22 | 0.40 | 0.58 | 1.25 | 0.57 | 2.72 | 0.89 | 0.69 | 0.76 | 0.37 | 1.99 | 0.45 | 8.78 | 0.68 |
| JSW | order Bacillales id 1674 | 26 | 0.20 | 0.45 | 0.66 | 1.22 | 0.50 | 2.97 | 0.88 | 0.02 | 0.64 | 0.98 | 1.02 | 0.29 | 3.59 | 0.98 | -0.39 | 1.36 | 0.78 | 0.68 | 0.05 | 9.69 | 0.88 |
| JSW | order Bacteroidales id 913 | 28 | -0.11 | 0.19 | 0.57 | 0.90 | 0.63 | 1.29 | 0.81 | 0.02 | 0.27 | 0.93 | 1.02 | 0.61 | 1.72 | 0.98 | 0.34 | 0.60 | 0.58 | 1.40 | 0.43 | 4.54 | 0.77 |
| JSW | order Bifidobacteriales id 432 | 28 | 0.20 | 0.24 | 0.41 | 1.22 | 0.76 | 1.94 | 0.71 | 0.20 | 0.30 | 0.51 | 1.22 | 0.68 | 2.20 | 0.85 | -0.79 | 0.71 | 0.28 | 0.46 | 0.11 | 1.84 | 0.68 |
| JSW | order Burkholderiales id 2874 | 28 | -0.20 | 0.19 | 0.31 | 0.82 | 0.56 | 1.20 | 0.71 | -0.27 | 0.25 | 0.28 | 0.76 | 0.47 | 1.24 | 0.60 | -0.62 | 0.50 | 0.22 | 0.54 | 0.20 | 1.42 | 0.68 |
| JSW | order Clostridiales id 1863 | 28 | 0.03 | 0.19 | 0.88 | 1.03 | 0.72 | 1.48 | 0.99 | 0.25 | 0.26 | 0.33 | 1.29 | 0.78 | 2.13 | 0.60 | 0.45 | 0.52 | 0.40 | 1.57 | 0.56 | 4.37 | 0.68 |
| JSW | order Coriobacteriales id 810 | 28 | 0.40 | 0.19 | 0.03 | 1.49 | 1.03 | 2.17 | 0.61 | 0.36 | 0.27 | 0.18 | 1.44 | 0.85 | 2.45 | 0.60 | 0.40 | 0.52 | 0.45 | 1.49 | 0.54 | 4.15 | 0.68 |
| JSW | order Desulfovibrionales id 3156 | 28 | -0.19 | 0.22 | 0.37 | 0.82 | 0.54 | 1.26 | 0.71 | -0.38 | 0.28 | 0.18 | 0.68 | 0.39 | 1.19 | 0.60 | -0.58 | 0.65 | 0.38 | 0.56 | 0.16 | 2.00 | 0.68 |
| JSW | order Enterobacteriales id 3468 | 28 | -0.40 | 0.22 | 0.06 | 0.67 | 0.44 | 1.02 | 0.61 | -0.62 | 0.31 | 0.04 | 0.54 | 0.29 | 0.99 | 0.45 | -0.68 | 0.58 | 0.26 | 0.51 | 0.16 | 1.60 | 0.68 |
| JSW | order Erysipelotrichales id 2148 | 28 | -0.22 | 0.21 | 0.30 | 0.80 | 0.53 | 1.22 | 0.71 | -0.29 | 0.26 | 0.27 | 0.75 | 0.45 | 1.25 | 0.60 | -0.36 | 0.50 | 0.48 | 0.70 | 0.26 | 1.87 | 0.68 |
| JSW | order Gastranaerophilales id 1591 | 28 | 0.45 | 0.37 | 0.22 | 1.58 | 0.77 | 3.24 | 0.71 | 1.00 | 0.47 | 0.03 | 2.71 | 1.07 | 6.84 | 0.45 | 1.31 | 0.96 | 0.18 | 3.71 | 0.57 | 24.26 | 0.68 |
| JSW | order Lactobacillales id 1800 | 28 | 0.01 | 0.19 | 0.97 | 1.01 | 0.69 | 1.46 | 0.99 | 0.07 | 0.27 | 0.78 | 1.08 | 0.63 | 1.83 | 0.98 | 0.41 | 0.52 | 0.44 | 1.50 | 0.54 | 4.18 | 0.68 |
| JSW | order Methanobacteriales id 120 | 26 | 0.38 | 0.44 | 0.39 | 1.46 | 0.62 | 3.45 | 0.71 | 0.19 | 0.63 | 0.76 | 1.21 | 0.36 | 4.13 | 0.98 | 0.61 | 1.28 | 0.64 | 1.83 | 0.15 | 22.52 | 0.80 |
| JSW | order MollicutesRF9 id 11579 | 28 | 0.22 | 0.28 | 0.43 | 1.25 | 0.72 | 2.16 | 0.71 | 0.48 | 0.35 | 0.17 | 1.62 | 0.81 | 3.26 | 0.60 | 0.92 | 0.73 | 0.22 | 2.52 | 0.60 | 10.58 | 0.68 |
| JSW | order NB1n id 3953 | 27 | 0.02 | 0.37 | 0.95 | 1.02 | 0.49 | 2.12 | 0.99 | 0.03 | 0.51 | 0.95 | 1.03 | 0.38 | 2.82 | 0.98 | -0.18 | 1.06 | 0.87 | 0.84 | 0.11 | 6.62 | 0.88 |
| JSW | order Pasteurellales id 3688 | 28 | 0.05 | 0.28 | 0.87 | 1.05 | 0.61 | 1.80 | 0.99 | -0.14 | 0.39 | 0.72 | 0.87 | 0.40 | 1.88 | 0.98 | -0.11 | 0.75 | 0.88 | 0.89 | 0.20 | 3.89 | 0.88 |
| JSW | order Rhodospirillales id 2667 | 28 | 0.27 | 0.31 | 0.39 | 1.30 | 0.71 | 2.39 | 0.71 | 0.38 | 0.39 | 0.33 | 1.46 | 0.68 | 3.11 | 0.60 | 0.94 | 0.73 | 0.21 | 2.55 | 0.62 | 10.59 | 0.68 |
| JSW | order Selenomonadales id 2165 | 28 | 0.00 | 0.19 | 0.99 | 1.00 | 0.70 | 1.44 | 0.99 | -0.03 | 0.26 | 0.90 | 0.97 | 0.58 | 1.62 | 0.98 | -0.10 | 0.51 | 0.85 | 0.91 | 0.33 | 2.49 | 0.88 |
| JSW | order Verrucomicrobiales id 4030 | 28 | -0.33 | 0.23 | 0.14 | 0.72 | 0.46 | 1.12 | 0.71 | -0.32 | 0.31 | 0.30 | 0.72 | 0.39 | 1.34 | 0.60 | -1.00 | 0.63 | 0.13 | 0.37 | 0.11 | 1.27 | 0.68 |
| JSW | order Victivallales id 2254 | 27 | -0.34 | 0.37 | 0.35 | 0.71 | 0.34 | 1.46 | 0.71 | -0.70 | 0.51 | 0.17 | 0.49 | 0.18 | 1.35 | 0.60 | -1.02 | 1.02 | 0.33 | 0.36 | 0.05 | 2.66 | 0.68 |
| JSW | family Acidaminococcaceae id 2166 | 28 | 0.20 | 0.22 | 0.37 | 1.22 | 0.79 | 1.90 | 0.73 | 0.18 | 0.31 | 0.57 | 1.20 | 0.65 | 2.22 | 0.87 | 0.07 | 0.61 | 0.91 | 1.07 | 0.33 | 3.53 | 0.91 |
| JSW | family Actinomycetaceae id 421 | 28 | 0.17 | 0.28 | 0.55 | 1.18 | 0.68 | 2.06 | 0.89 | 0.22 | 0.41 | 0.60 | 1.24 | 0.55 | 2.79 | 0.87 | 0.72 | 0.76 | 0.36 | 2.05 | 0.46 | 9.12 | 0.80 |
| JSW | family Alcaligenaceae id 2875 | 28 | -0.18 | 0.19 | 0.34 | 0.83 | 0.57 | 1.22 | 0.73 | -0.25 | 0.25 | 0.31 | 0.78 | 0.48 | 1.26 | 0.85 | -0.41 | 0.48 | 0.40 | 0.66 | 0.26 | 1.69 | 0.80 |
| JSW | family Bacteroidaceae id 917 | 28 | -0.25 | 0.19 | 0.18 | 0.78 | 0.54 | 1.12 | 0.73 | -0.01 | 0.27 | 0.98 | 0.99 | 0.58 | 1.69 | 0.98 | 0.12 | 0.52 | 0.81 | 1.13 | 0.41 | 3.15 | 0.87 |

| | | | | | | | | | | | | | | | | | | | | | | | |
|---|---|---|---|---|---|---|---|---|---|---|---|---|---|---|---|---|---|---|---|---|---|---|---|
| JSW | family BacteroidalesS24 7group id 11173 | 28 | 0.14 | 0.28 | 0.62 | 1.15 | 0.66 | 2.00 | 0.89 | 0.33 | 0.42 | 0.43 | 1.39 | 0.62 | 3.14 | 0.85 | 0.95 | 0.90 | 0.30 | 2.57 | 0.44 | 15.05 | 0.80 |
| JSW | family Bifidobacteriaceae id 433 | 28 | 0.20 | 0.24 | 0.41 | 1.22 | 0.76 | 1.94 | 0.73 | 0.20 | 0.30 | 0.50 | 1.22 | 0.68 | 2.18 | 0.85 | -0.79 | 0.70 | 0.27 | 0.46 | 0.12 | 1.80 | 0.80 |
| JSW | family Christensenellaceae id 1866 | 28 | -0.11 | 0.21 | 0.61 | 0.90 | 0.59 | 1.36 | 0.89 | -0.21 | 0.28 | 0.45 | 0.81 | 0.47 | 1.39 | 0.85 | -0.28 | 0.54 | 0.60 | 0.75 | 0.26 | 2.15 | 0.80 |
| JSW | family Clostridiaceae1 id 1869 | 28 | -0.24 | 0.22 | 0.27 | 0.79 | 0.51 | 1.21 | 0.73 | -0.08 | 0.30 | 0.80 | 0.93 | 0.51 | 1.67 | 0.88 | 0.26 | 0.58 | 0.65 | 1.30 | 0.42 | 4.04 | 0.80 |
| JSW | family ClostridialesvadinBB60group id 11286 | 28 | -0.01 | 0.27 | 0.98 | 0.99 | 0.58 | 1.69 | 0.98 | 0.04 | 0.36 | 0.91 | 1.04 | 0.52 | 2.10 | 0.94 | -0.27 | 0.69 | 0.70 | 0.77 | 0.20 | 2.95 | 0.83 |
| JSW | family Coriobacteriaceae id 811 | 28 | 0.40 | 0.19 | 0.03 | 1.49 | 1.03 | 2.17 | 0.71 | 0.36 | 0.27 | 0.18 | 1.44 | 0.84 | 2.46 | 0.85 | 0.40 | 0.56 | 0.48 | 1.49 | 0.50 | 4.49 | 0.80 |
| JSW | family Defluviitaleaceae id 1924 | 28 | 0.13 | 0.28 | 0.64 | 1.14 | 0.66 | 1.96 | 0.89 | 0.34 | 0.38 | 0.37 | 1.40 | 0.67 | 2.95 | 0.85 | 1.05 | 0.76 | 0.17 | 2.87 | 0.65 | 12.61 | 0.80 |
| JSW | family Desulfovibrionaceae id 3169 | 28 | -0.20 | 0.22 | 0.37 | 0.82 | 0.53 | 1.26 | 0.73 | -0.40 | 0.30 | 0.19 | 0.67 | 0.37 | 1.21 | 0.85 | -0.55 | 0.58 | 0.35 | 0.58 | 0.19 | 1.79 | 0.80 |
| JSW | family Enterobacteriaceae id 3469 | 28 | -0.40 | 0.22 | 0.06 | 0.67 | 0.44 | 1.02 | 0.71 | -0.62 | 0.31 | 0.04 | 0.54 | 0.29 | 0.98 | 0.85 | -0.68 | 0.67 | 0.32 | 0.51 | 0.14 | 1.88 | 0.80 |
| JSW | family Erysipelotrichaceae id 2149 | 28 | -0.22 | 0.21 | 0.30 | 0.80 | 0.53 | 1.22 | 0.73 | -0.29 | 0.27 | 0.28 | 0.75 | 0.44 | 1.27 | 0.85 | -0.36 | 0.48 | 0.46 | 0.70 | 0.27 | 1.80 | 0.80 |
| JSW | family FamilyXI id 1936 | 22 | -0.05 | 0.59 | 0.94 | 0.95 | 0.30 | 3.02 | 0.98 | -0.20 | 0.71 | 0.78 | 0.82 | 0.20 | 3.31 | 0.88 | 1.18 | 1.72 | 0.50 | 3.26 | 0.11 | 94.56 | 0.80 |
| JSW | family FamilyXIII id 1957 | 28 | 0.10 | 0.23 | 0.67 | 1.10 | 0.70 | 1.73 | 0.89 | 0.41 | 0.30 | 0.17 | 1.51 | 0.84 | 2.71 | 0.85 | 0.99 | 0.68 | 0.16 | 2.69 | 0.71 | 10.21 | 0.80 |
| JSW | family Lachnospiraceae id 1987 | 28 | -0.31 | 0.19 | 0.09 | 0.73 | 0.51 | 1.05 | 0.73 | -0.45 | 0.26 | 0.08 | 0.64 | 0.38 | 1.06 | 0.85 | -0.46 | 0.55 | 0.41 | 0.63 | 0.22 | 1.85 | 0.80 |
| JSW | family Lactobacillaceae id 1836 | 27 | 0.09 | 0.32 | 0.79 | 1.09 | 0.58 | 2.03 | 0.93 | 0.41 | 0.41 | 0.31 | 1.51 | 0.68 | 3.35 | 0.85 | 0.38 | 0.79 | 0.64 | 1.46 | 0.31 | 6.86 | 0.80 |
| JSW | family Methanobacteriaceae id 121 | 26 | 0.38 | 0.44 | 0.39 | 1.46 | 0.62 | 3.45 | 0.73 | 0.19 | 0.61 | 0.75 | 1.21 | 0.37 | 4.02 | 0.88 | 0.61 | 1.23 | 0.63 | 1.83 | 0.17 | 20.30 | 0.80 |
| JSW | family Oxalobacteraceae id 2966 | 27 | 0.05 | 0.38 | 0.90 | 1.05 | 0.50 | 2.22 | 0.98 | -0.12 | 0.49 | 0.80 | 0.88 | 0.34 | 2.32 | 0.88 | -0.93 | 1.14 | 0.42 | 0.39 | 0.04 | 3.65 | 0.80 |
| JSW | family Pasteurellaceae id 3689 | 28 | 0.05 | 0.28 | 0.87 | 1.05 | 0.61 | 1.80 | 0.98 | -0.14 | 0.37 | 0.71 | 0.87 | 0.42 | 1.80 | 0.88 | -0.11 | 0.76 | 0.88 | 0.89 | 0.20 | 3.98 | 0.91 |
| JSW | family Peptococcaceae id 2024 | 28 | 0.26 | 0.26 | 0.32 | 1.29 | 0.78 | 2.14 | 0.73 | 0.28 | 0.34 | 0.41 | 1.32 | 0.68 | 2.54 | 0.85 | 0.16 | 0.64 | 0.80 | 1.17 | 0.34 | 4.09 | 0.87 |
| JSW | family Peptostreptococcaceae id 2042 | 28 | 0.26 | 0.21 | 0.22 | 1.29 | 0.86 | 1.95 | 0.73 | 0.32 | 0.28 | 0.26 | 1.37 | 0.79 | 2.40 | 0.85 | 0.44 | 0.60 | 0.47 | 1.55 | 0.48 | 4.99 | 0.80 |
| JSW | family Porphyromonadaceae id 943 | 28 | 0.00 | 0.19 | 0.98 | 1.00 | 0.70 | 1.45 | 0.98 | 0.06 | 0.26 | 0.82 | 1.06 | 0.64 | 1.75 | 0.88 | 0.25 | 0.50 | 0.61 | 1.29 | 0.49 | 3.42 | 0.80 |
| JSW | family Prevotellaceae id 960 | 28 | -0.09 | 0.28 | 0.75 | 0.91 | 0.53 | 1.58 | 0.92 | -0.19 | 0.32 | 0.55 | 0.83 | 0.44 | 1.55 | 0.87 | -0.66 | 0.70 | 0.36 | 0.52 | 0.13 | 2.05 | 0.80 |
| JSW | family Rhodospirillaceae id 2717 | 28 | 0.25 | 0.30 | 0.41 | 1.28 | 0.71 | 2.32 | 0.73 | 0.28 | 0.40 | 0.48 | 1.33 | 0.60 | 2.91 | 0.85 | 0.95 | 0.80 | 0.24 | 2.58 | 0.54 | 12.26 | 0.80 |

| | | | | | | | | | | | | | | | | | | | | | | |
|---|---|---|---|---|---|---|---|---|---|---|---|---|---|---|---|---|---|---|---|---|---|---|
| JSW | family Rikenellaceae id 967 | 28 | 0.22 | 0.19 | 0.24 | 1.25 | 0.86 | 1.81 | 0.73 | -0.06 | 0.27 | 0.83 | 0.94 | 0.55 | 1.61 | 0.88 | -0.33 | 0.56 | 0.56 | 0.72 | 0.24 | 2.16 | 0.80 |
| JSW | family Ruminococcaceae id 2050 | 28 | 0.39 | 0.21 | 0.07 | 1.47 | 0.97 | 2.22 | 0.71 | 0.26 | 0.27 | 0.34 | 1.30 | 0.76 | 2.20 | 0.85 | 0.25 | 0.51 | 0.63 | 1.29 | 0.47 | 3.49 | 0.80 |
| JSW | family Streptococcaceae id 1850 | 28 | -0.08 | 0.19 | 0.69 | 0.93 | 0.63 | 1.36 | 0.89 | -0.13 | 0.27 | 0.63 | 0.88 | 0.51 | 1.50 | 0.88 | -0.33 | 0.56 | 0.56 | 0.72 | 0.24 | 2.15 | 0.80 |
| JSW | family Veillonellaceae id 2172 | 28 | -0.10 | 0.20 | 0.62 | 0.91 | 0.61 | 1.34 | 0.89 | -0.19 | 0.27 | 0.49 | 0.83 | 0.49 | 1.41 | 0.85 | -0.20 | 0.56 | 0.72 | 0.82 | 0.27 | 2.44 | 0.83 |
| JSW | family Verrucomicrobiaceae id 4036 | 28 | -0.34 | 0.23 | 0.14 | 0.71 | 0.46 | 1.12 | 0.73 | -0.32 | 0.30 | 0.29 | 0.72 | 0.40 | 1.31 | 0.85 | -1.00 | 0.63 | 0.12 | 0.37 | 0.11 | 1.27 | 0.80 |
| JSW | family Victivallaceae id 2255 | 27 | 0.38 | 0.41 | 0.35 | 1.46 | 0.66 | 3.22 | 0.73 | 0.55 | 0.56 | 0.32 | 1.74 | 0.58 | 5.23 | 0.85 | 0.56 | 1.22 | 0.65 | 1.76 | 0.16 | 19.31 | 0.80 |
| JSW | genus Clostridiuminnocuumgroup id 14397 | 27 | 0.67 | 0.39 | 0.09 | 1.95 | 0.90 | 4.20 | 0.88 | 0.22 | 0.54 | 0.68 | 1.25 | 0.43 | 3.57 | 0.94 | -0.37 | 1.03 | 0.72 | 0.69 | 0.09 | 5.23 | 0.96 |
| JSW | genus Eubacteriumbrachygroup id 11296 | 27 | 1.05 | 0.40 | 0.01 | 2.85 | 1.30 | 6.25 | 0.51 | 0.70 | 0.55 | 0.20 | 2.02 | 0.69 | 5.93 | 0.94 | 0.00 | 1.14 | 1.00 | 1.00 | 0.11 | 9.35 | 1.00 |
| JSW | genus Eubacteriumcoprostanoligenesgroup id 11375 | 28 | 0.16 | 0.24 | 0.52 | 1.17 | 0.73 | 1.88 | 0.88 | -0.06 | 0.30 | 0.83 | 0.94 | 0.53 | 1.67 | 0.95 | 0.76 | 0.64 | 0.24 | 2.14 | 0.61 | 7.51 | 0.96 |
| JSW | genus Eubacteriumeligensgroup id 14372 | 28 | 0.16 | 0.21 | 0.43 | 1.18 | 0.79 | 1.77 | 0.88 | -0.01 | 0.28 | 0.98 | 0.99 | 0.57 | 1.73 | 0.99 | -0.46 | 0.60 | 0.45 | 0.63 | 0.20 | 2.04 | 0.96 |
| JSW | genus Eubacteriumfissicatenagroup id 14373 | 27 | -0.45 | 0.44 | 0.31 | 0.64 | 0.27 | 1.53 | 0.88 | -0.77 | 0.60 | 0.20 | 0.47 | 0.14 | 1.51 | 0.94 | -1.41 | 1.28 | 0.28 | 0.24 | 0.02 | 3.02 | 0.96 |
| JSW | genus Eubacteriumhalliigroup id 11338 | 28 | 0.35 | 0.19 | 0.07 | 1.42 | 0.97 | 2.08 | 0.88 | 0.35 | 0.28 | 0.21 | 1.42 | 0.82 | 2.45 | 0.94 | 0.29 | 0.52 | 0.58 | 1.34 | 0.48 | 3.69 | 0.96 |
| JSW | genus Eubacteriumnodatumgroup id 11297 | 26 | -0.51 | 0.48 | 0.29 | 0.60 | 0.23 | 1.53 | 0.88 | -0.03 | 0.64 | 0.96 | 0.97 | 0.28 | 3.35 | 0.99 | 0.21 | 1.28 | 0.87 | 1.23 | 0.10 | 15.05 | 0.97 |
| JSW | genus Eubacteriumoxidoreducensgroup id 11339 | 27 | -0.27 | 0.35 | 0.44 | 0.77 | 0.39 | 1.51 | 0.88 | -0.50 | 0.48 | 0.30 | 0.61 | 0.24 | 1.55 | 0.94 | -0.49 | 0.92 | 0.59 | 0.61 | 0.10 | 3.68 | 0.96 |

| | | | | | | | | | | | | | | | | | | | | | | | |
|---|---|---|---|---|---|---|---|---|---|---|---|---|---|---|---|---|---|---|---|---|---|---|---|
| JSW | genus Eubacteriumrectalegroup id 14374 | 28 | -0.21 | 0.21 | 0.32 | 0.81 | 0.54 | 1.22 | 0.88 | -0.32 | 0.27 | 0.23 | 0.72 | 0.42 | 1.23 | 0.94 | -0.46 | 0.49 | 0.35 | 0.63 | 0.24 | 1.64 | 0.96 |
| JSW | genus Eubacteriumruminantiumgroup id 11340 | 28 | -0.14 | 0.33 | 0.67 | 0.87 | 0.45 | 1.66 | 0.88 | -0.22 | 0.43 | 0.61 | 0.80 | 0.35 | 1.85 | 0.94 | -1.75 | 1.03 | 0.10 | 0.17 | 0.02 | 1.30 | 0.96 |
| JSW | genus Eubacteriumventriosumgroup id 11341 | 28 | -0.04 | 0.20 | 0.85 | 0.96 | 0.65 | 1.43 | 0.93 | -0.21 | 0.29 | 0.47 | 0.81 | 0.46 | 1.43 | 0.94 | -0.63 | 0.55 | 0.26 | 0.53 | 0.18 | 1.56 | 0.96 |
| JSW | genus Eubacteriumxylanophilumgroup id 14375 | 28 | -0.34 | 0.22 | 0.11 | 0.71 | 0.46 | 1.08 | 0.88 | -0.52 | 0.30 | 0.08 | 0.60 | 0.33 | 1.07 | 0.94 | -0.79 | 0.64 | 0.22 | 0.45 | 0.13 | 1.58 | 0.96 |
| JSW | genus Ruminococcusgauvreauiigroup id 11342 | 28 | -0.13 | 0.22 | 0.57 | 0.88 | 0.57 | 1.36 | 0.88 | -0.10 | 0.31 | 0.74 | 0.90 | 0.49 | 1.65 | 0.94 | -0.73 | 0.68 | 0.29 | 0.48 | 0.13 | 1.82 | 0.96 |
| JSW | genus Ruminococcusgnavusgroup id 14376 | 27 | -0.71 | 0.36 | 0.05 | 0.49 | 0.24 | 0.99 | 0.88 | -1.20 | 0.46 | 0.01 | 0.30 | 0.12 | 0.75 | 0.58 | -1.50 | 0.87 | 0.10 | 0.22 | 0.04 | 1.24 | 0.96 |
| JSW | genus Ruminococcustorquesgroup id 14377 | 28 | 0.11 | 0.19 | 0.57 | 1.12 | 0.76 | 1.64 | 0.88 | 0.11 | 0.26 | 0.68 | 1.12 | 0.67 | 1.87 | 0.94 | -0.05 | 0.56 | 0.93 | 0.95 | 0.32 | 2.86 | 0.98 |
| JSW | genus Actinomyces id 423 | 28 | 0.17 | 0.29 | 0.56 | 1.18 | 0.67 | 2.08 | 0.88 | 0.28 | 0.40 | 0.49 | 1.32 | 0.60 | 2.91 | 0.94 | 0.41 | 0.82 | 0.62 | 1.51 | 0.30 | 7.54 | 0.96 |
| JSW | genus Adlercreutzia id 812 | 28 | 0.55 | 0.36 | 0.12 | 1.74 | 0.86 | 3.49 | 0.88 | 0.52 | 0.42 | 0.22 | 1.69 | 0.74 | 3.87 | 0.94 | 0.92 | 0.91 | 0.32 | 2.52 | 0.42 | 15.04 | 0.96 |
| JSW | genus Akkermansia id 4037 | 28 | -0.34 | 0.23 | 0.14 | 0.71 | 0.45 | 1.12 | 0.88 | -0.35 | 0.30 | 0.25 | 0.70 | 0.39 | 1.28 | 0.94 | -1.01 | 0.66 | 0.14 | 0.37 | 0.10 | 1.33 | 0.96 |
| JSW | genus Alistipes id 968 | 28 | 0.13 | 0.19 | 0.51 | 1.13 | 0.78 | 1.66 | 0.88 | -0.14 | 0.27 | 0.61 | 0.87 | 0.51 | 1.48 | 0.94 | -0.42 | 0.57 | 0.47 | 0.66 | 0.21 | 2.01 | 0.96 |
| JSW | genus Allisonella id 2174 | 23 | -0.76 | 0.48 | 0.11 | 0.47 | 0.18 | 1.19 | 0.88 | -0.52 | 0.63 | 0.41 | 0.59 | 0.17 | 2.06 | 0.94 | -0.33 | 1.24 | 0.79 | 0.72 | 0.06 | 8.10 | 0.96 |
| JSW | genus Alloprevotella id 961 | 14 | 0.16 | 0.74 | 0.83 | 1.17 | 0.28 | 4.95 | 0.93 | 0.75 | 0.89 | 0.40 | 2.12 | 0.37 | 12.05 | 0.94 | 1.20 | 1.45 | 0.42 | 3.32 | 0.19 | 57.01 | 0.96 |
| JSW | genus Anaerofilum id 2053 | 27 | -0.17 | 0.40 | 0.67 | 0.84 | 0.38 | 1.85 | 0.88 | -0.01 | 0.55 | 0.98 | 0.99 | 0.33 | 2.91 | 0.99 | 0.57 | 1.12 | 0.61 | 1.77 | 0.20 | 15.75 | 0.96 |
| JSW | genus Anaerostipes id 1991 | 28 | -0.10 | 0.22 | 0.66 | 0.91 | 0.59 | 1.40 | 0.88 | -0.08 | 0.29 | 0.77 | 0.92 | 0.52 | 1.62 | 0.94 | 0.08 | 0.56 | 0.89 | 1.08 | 0.36 | 3.24 | 0.97 |

| | | | | | | | | | | | | | | | | | | | | | | | |
|---|---|---|---|---|---|---|---|---|---|---|---|---|---|---|---|---|---|---|---|---|---|---|---|
| JSW | genus Anaerotruncus id 2054 | 28 | -0.23 | 0.23 | 0.33 | 0.80 | 0.51 | 1.25 | 0.88 | -0.22 | 0.31 | 0.47 | 0.80 | 0.44 | 1.46 | 0.94 | 0.54 | 0.70 | 0.45 | 1.71 | 0.43 | 6.75 | 0.96 |
| JSW | genus Bacteroides id 918 | 28 | -0.25 | 0.19 | 0.18 | 0.78 | 0.54 | 1.12 | 0.88 | -0.01 | 0.26 | 0.98 | 0.99 | 0.60 | 1.65 | 0.99 | 0.12 | 0.55 | 0.82 | 1.13 | 0.38 | 3.33 | 0.96 |
| JSW | genus Barnesiella id 944 | 28 | 0.35 | 0.23 | 0.12 | 1.42 | 0.92 | 2.22 | 0.88 | 0.53 | 0.30 | 0.07 | 1.70 | 0.95 | 3.04 | 0.94 | 0.20 | 0.60 | 0.74 | 1.22 | 0.38 | 3.95 | 0.96 |
| JSW | genus Bifidobacterium id 436 | 28 | 0.20 | 0.23 | 0.39 | 1.22 | 0.78 | 1.90 | 0.88 | 0.19 | 0.30 | 0.53 | 1.21 | 0.67 | 2.20 | 0.94 | -0.66 | 0.63 | 0.30 | 0.51 | 0.15 | 1.78 | 0.96 |
| JSW | genus Bilophila id 3170 | 28 | -0.22 | 0.22 | 0.31 | 0.80 | 0.52 | 1.23 | 0.88 | -0.21 | 0.31 | 0.50 | 0.81 | 0.44 | 1.49 | 0.94 | 0.58 | 0.68 | 0.40 | 1.78 | 0.47 | 6.78 | 0.96 |
| JSW | genus Blautia id 1992 | 28 | -0.14 | 0.19 | 0.46 | 0.87 | 0.60 | 1.25 | 0.88 | 0.10 | 0.25 | 0.70 | 1.10 | 0.67 | 1.80 | 0.94 | 0.24 | 0.49 | 0.63 | 1.27 | 0.48 | 3.36 | 0.96 |
| JSW | genus Butyricicoccus id 2055 | 28 | 0.06 | 0.19 | 0.75 | 1.06 | 0.73 | 1.55 | 0.92 | 0.03 | 0.26 | 0.92 | 1.03 | 0.62 | 1.70 | 0.97 | 0.11 | 0.52 | 0.83 | 1.12 | 0.40 | 3.10 | 0.96 |
| JSW | genus Butyricimonas id 945 | 28 | 0.04 | 0.24 | 0.87 | 1.04 | 0.65 | 1.67 | 0.93 | -0.15 | 0.32 | 0.64 | 0.86 | 0.46 | 1.60 | 0.94 | -0.20 | 0.62 | 0.75 | 0.82 | 0.24 | 2.78 | 0.96 |
| JSW | genus Butyrivibrio id 1993 | 26 | 0.37 | 0.51 | 0.46 | 1.45 | 0.54 | 3.92 | 0.88 | 0.68 | 0.67 | 0.31 | 1.98 | 0.54 | 7.28 | 0.94 | 1.00 | 1.43 | 0.49 | 2.72 | 0.17 | 44.49 | 0.96 |
| JSW | genus CandidatusSoleaferrea id 11350 | 27 | -0.06 | 0.39 | 0.88 | 0.94 | 0.44 | 2.03 | 0.93 | -0.27 | 0.46 | 0.56 | 0.77 | 0.31 | 1.89 | 0.94 | -0.61 | 0.91 | 0.51 | 0.55 | 0.09 | 3.26 | 0.96 |
| JSW | genus Catenibacterium id 2153 | 23 | -0.47 | 0.48 | 0.32 | 0.62 | 0.24 | 1.59 | 0.88 | -0.45 | 0.61 | 0.46 | 0.64 | 0.19 | 2.10 | 0.94 | -0.59 | 1.18 | 0.62 | 0.56 | 0.06 | 5.57 | 0.96 |
| JSW | genus ChristensenellaceaeR 7group id 11283 | 28 | -0.02 | 0.22 | 0.91 | 0.98 | 0.64 | 1.49 | 0.94 | -0.24 | 0.28 | 0.39 | 0.79 | 0.46 | 1.36 | 0.94 | -0.26 | 0.50 | 0.61 | 0.77 | 0.29 | 2.06 | 0.96 |
| JSW | genus Clostridiumsensustricto1 id 1873 | 28 | -0.24 | 0.21 | 0.26 | 0.79 | 0.52 | 1.19 | 0.88 | -0.09 | 0.30 | 0.76 | 0.91 | 0.50 | 1.64 | 0.94 | 0.25 | 0.60 | 0.68 | 1.28 | 0.40 | 4.14 | 0.96 |
| JSW | genus Collinsella id 815 | 28 | 0.33 | 0.21 | 0.12 | 1.39 | 0.92 | 2.09 | 0.88 | 0.50 | 0.29 | 0.08 | 1.65 | 0.94 | 2.91 | 0.94 | 0.70 | 0.61 | 0.26 | 2.01 | 0.61 | 6.60 | 0.96 |
| JSW | genus Coprobacter id 949 | 27 | -0.13 | 0.31 | 0.67 | 0.88 | 0.48 | 1.60 | 0.88 | -0.58 | 0.43 | 0.18 | 0.56 | 0.24 | 1.32 | 0.94 | -0.81 | 0.92 | 0.39 | 0.45 | 0.07 | 2.69 | 0.96 |
| JSW | genus Coprococcus1 id 11301 | 28 | 0.23 | 0.21 | 0.28 | 1.26 | 0.83 | 1.92 | 0.88 | -0.08 | 0.28 | 0.79 | 0.93 | 0.53 | 1.62 | 0.94 | -0.10 | 0.53 | 0.85 | 0.91 | 0.32 | 2.55 | 0.96 |
| JSW | genus Coprococcus2 id 11302 | 28 | -0.10 | 0.27 | 0.72 | 0.91 | 0.54 | 1.53 | 0.91 | 0.40 | 0.34 | 0.25 | 1.49 | 0.76 | 2.92 | 0.94 | 0.37 | 0.69 | 0.60 | 1.45 | 0.37 | 5.61 | 0.96 |
| JSW | genus Coprococcus3 id 11303 | 28 | 0.14 | 0.20 | 0.49 | 1.15 | 0.78 | 1.70 | 0.88 | 0.21 | 0.30 | 0.48 | 1.24 | 0.69 | 2.22 | 0.94 | 0.35 | 0.58 | 0.55 | 1.42 | 0.46 | 4.41 | 0.96 |
| JSW | genus DefluviitaleaceaeUCG011 id 11287 | 28 | 0.15 | 0.28 | 0.58 | 1.16 | 0.68 | 2.00 | 0.88 | 0.35 | 0.39 | 0.37 | 1.42 | 0.66 | 3.02 | 0.94 | 1.12 | 0.78 | 0.16 | 3.06 | 0.67 | 14.01 | 0.96 |
| JSW | genus Desulfovibrio id 3173 | 28 | -0.04 | 0.29 | 0.88 | 0.96 | 0.55 | 1.67 | 0.93 | -0.39 | 0.39 | 0.32 | 0.67 | 0.31 | 1.46 | 0.94 | -0.98 | 0.88 | 0.27 | 0.38 | 0.07 | 2.09 | 0.96 |
| JSW | genus Dialister id 2183 | 28 | -0.22 | 0.27 | 0.42 | 0.80 | 0.47 | 1.37 | 0.88 | -0.23 | 0.34 | 0.50 | 0.79 | 0.40 | 1.55 | 0.94 | -0.26 | 0.67 | 0.70 | 0.77 | 0.21 | 2.85 | 0.96 |
| JSW | genus Dorea id 1997 | 28 | 0.05 | 0.19 | 0.79 | 1.05 | 0.73 | 1.53 | 0.92 | 0.07 | 0.26 | 0.77 | 1.08 | 0.65 | 1.80 | 0.94 | -0.58 | 0.60 | 0.34 | 0.56 | 0.17 | 1.80 | 0.96 |

| | | | | | | | | | | | | | | | | | | | | | | | |
|---|---|---|---|---|---|---|---|---|---|---|---|---|---|---|---|---|---|---|---|---|---|---|---|
| JSW | genus Eggerthella id 819 | 27 | 0.21 | 0.35 | 0.55 | 1.23 | 0.62 | 2.45 | 0.88 | -0.03 | 0.48 | 0.95 | 0.97 | 0.38 | 2.47 | 0.99 | -0.09 | 0.94 | 0.92 | 0.91 | 0.14 | 5.80 | 0.98 |
| JSW | genus Eisenbergiella id 11304 | 27 | -0.19 | 0.42 | 0.66 | 0.83 | 0.37 | 1.89 | 0.88 | -0.52 | 0.49 | 0.29 | 0.60 | 0.23 | 1.57 | 0.94 | -0.80 | 0.94 | 0.40 | 0.45 | 0.07 | 2.81 | 0.96 |
| JSW | genus Enterorhabdus id 820 | 28 | 0.27 | 0.30 | 0.37 | 1.31 | 0.73 | 2.36 | 0.88 | -0.18 | 0.42 | 0.67 | 0.84 | 0.37 | 1.92 | 0.94 | -0.65 | 0.86 | 0.46 | 0.52 | 0.10 | 2.85 | 0.96 |
| JSW | genus Erysipelatoclostridium id 11381 | 28 | -0.18 | 0.25 | 0.47 | 0.83 | 0.51 | 1.37 | 0.88 | -0.12 | 0.35 | 0.73 | 0.89 | 0.44 | 1.77 | 0.94 | -0.35 | 0.82 | 0.67 | 0.70 | 0.14 | 3.54 | 0.96 |
| JSW | genus ErysipelotrichaceaeUCG003 id 11384 | 28 | -0.24 | 0.22 | 0.27 | 0.79 | 0.52 | 1.21 | 0.88 | -0.19 | 0.29 | 0.51 | 0.83 | 0.47 | 1.45 | 0.94 | -0.24 | 0.57 | 0.67 | 0.78 | 0.25 | 2.42 | 0.96 |
| JSW | genus Escherichia Shigella id 3504 | 28 | -0.26 | 0.24 | 0.29 | 0.77 | 0.48 | 1.24 | 0.88 | -0.28 | 0.34 | 0.41 | 0.76 | 0.39 | 1.47 | 0.94 | -0.42 | 0.72 | 0.56 | 0.66 | 0.16 | 2.69 | 0.96 |
| JSW | genus Faecalibacterium id 2057 | 28 | 0.05 | 0.19 | 0.79 | 1.05 | 0.73 | 1.52 | 0.92 | 0.04 | 0.26 | 0.88 | 1.04 | 0.62 | 1.73 | 0.96 | 0.10 | 0.50 | 0.84 | 1.11 | 0.42 | 2.96 | 0.96 |
| JSW | genus FamilyXIIIAD3011group id 11293 | 28 | 0.16 | 0.22 | 0.47 | 1.17 | 0.77 | 1.78 | 0.88 | 0.20 | 0.30 | 0.51 | 1.22 | 0.68 | 2.20 | 0.94 | 0.35 | 0.62 | 0.58 | 1.42 | 0.42 | 4.78 | 0.96 |
| JSW | genus FamilyXIIIUCG001 id 11294 | 28 | -0.17 | 0.24 | 0.48 | 0.84 | 0.53 | 1.35 | 0.88 | 0.16 | 0.33 | 0.63 | 1.17 | 0.61 | 2.24 | 0.94 | 0.43 | 0.73 | 0.57 | 1.53 | 0.36 | 6.44 | 0.96 |
| JSW | genus Flavonifractor id 2059 | 28 | 0.10 | 0.26 | 0.70 | 1.11 | 0.66 | 1.86 | 0.90 | 0.16 | 0.34 | 0.64 | 1.18 | 0.60 | 2.30 | 0.94 | 0.24 | 0.61 | 0.69 | 1.28 | 0.39 | 4.21 | 0.96 |
| JSW | genus Fusicatenibacter id 11305 | 28 | -0.13 | 0.21 | 0.52 | 0.88 | 0.59 | 1.31 | 0.88 | -0.03 | 0.29 | 0.91 | 0.97 | 0.55 | 1.70 | 0.97 | 0.03 | 0.56 | 0.96 | 1.03 | 0.34 | 3.11 | 0.98 |
| JSW | genus Gordonibacter id 821 | 27 | 0.39 | 0.41 | 0.35 | 1.47 | 0.66 | 3.30 | 0.88 | 0.44 | 0.58 | 0.45 | 1.55 | 0.50 | 4.84 | 0.94 | 0.75 | 1.17 | 0.52 | 2.12 | 0.22 | 20.86 | 0.96 |
| JSW | genus Haemophilus id 3698 | 28 | 0.11 | 0.26 | 0.68 | 1.12 | 0.67 | 1.87 | 0.89 | 0.15 | 0.37 | 0.69 | 1.16 | 0.57 | 2.37 | 0.94 | 0.67 | 0.74 | 0.37 | 1.96 | 0.46 | 8.41 | 0.96 |
| JSW | genus Holdemanella id 11393 | 28 | -0.08 | 0.29 | 0.77 | 0.92 | 0.52 | 1.63 | 0.92 | 0.00 | 0.41 | 1.00 | 1.00 | 0.45 | 2.22 | 1.00 | 0.05 | 0.85 | 0.96 | 1.05 | 0.20 | 5.50 | 0.98 |
| JSW | genus Holdemania id 2157 | 27 | -0.15 | 0.26 | 0.57 | 0.86 | 0.51 | 1.45 | 0.88 | -0.17 | 0.38 | 0.66 | 0.84 | 0.40 | 1.78 | 0.94 | 0.02 | 0.74 | 0.98 | 1.02 | 0.24 | 4.38 | 0.98 |
| JSW | genus Howardella id 2000 | 27 | 0.30 | 0.41 | 0.47 | 1.35 | 0.60 | 3.01 | 0.88 | 0.60 | 0.60 | 0.31 | 1.83 | 0.56 | 5.92 | 0.94 | 1.34 | 1.16 | 0.26 | 3.83 | 0.40 | 37.10 | 0.96 |
| JSW | genus Hungatella id 11306 | 27 | 0.36 | 0.39 | 0.36 | 1.44 | 0.67 | 3.09 | 0.88 | 0.87 | 0.54 | 0.11 | 2.39 | 0.83 | 6.90 | 0.94 | 1.14 | 1.02 | 0.27 | 3.11 | 0.42 | 22.87 | 0.96 |
| JSW | genus Intestinibacter id 11345 | 28 | 0.07 | 0.23 | 0.76 | 1.07 | 0.69 | 1.67 | 0.92 | -0.21 | 0.31 | 0.50 | 0.81 | 0.44 | 1.50 | 0.94 | -0.38 | 0.65 | 0.56 | 0.68 | 0.19 | 2.44 | 0.96 |
| JSW | genus Intestinimonas id 2062 | 28 | 0.41 | 0.23 | 0.08 | 1.50 | 0.96 | 2.35 | 0.88 | 0.08 | 0.30 | 0.80 | 1.08 | 0.60 | 1.96 | 0.94 | -0.30 | 0.60 | 0.63 | 0.74 | 0.23 | 2.41 | 0.96 |
| JSW | genus Lachnoclostridium id 11308 | 28 | -0.38 | 0.19 | 0.05 | 0.69 | 0.48 | 0.99 | 0.88 | -0.42 | 0.27 | 0.11 | 0.66 | 0.39 | 1.11 | 0.94 | -0.82 | 0.55 | 0.15 | 0.44 | 0.15 | 1.29 | 0.96 |
| JSW | genus Lachnospira id 2004 | 28 | -0.36 | 0.20 | 0.07 | 0.70 | 0.48 | 1.03 | 0.88 | -0.09 | 0.27 | 0.74 | 0.92 | 0.54 | 1.54 | 0.94 | 0.14 | 0.50 | 0.79 | 1.15 | 0.43 | 3.03 | 0.96 |

| | | | | | | | | | | | | | | | | | | | | | | | |
|---|---|---|---|---|---|---|---|---|---|---|---|---|---|---|---|---|---|---|---|---|---|---|---|
| JSW | genus LachnospiraceaeFCS020group id 11314 | 28 | -0.30 | 0.23 | 0.20 | 0.74 | 0.47 | 1.17 | 0.88 | -0.33 | 0.30 | 0.28 | 0.72 | 0.40 | 1.30 | 0.94 | -0.38 | 0.64 | 0.56 | 0.68 | 0.20 | 2.40 | 0.96 |
| JSW | genus LachnospiraceaeNC2004group id 11316 | 27 | 0.22 | 0.32 | 0.49 | 1.24 | 0.67 | 2.33 | 0.88 | 0.29 | 0.42 | 0.49 | 1.34 | 0.59 | 3.08 | 0.94 | 0.30 | 0.82 | 0.72 | 1.35 | 0.27 | 6.67 | 0.96 |
| JSW | genus LachnospiraceaeND3007group id 11317 | 28 | -0.18 | 0.20 | 0.37 | 0.83 | 0.56 | 1.24 | 0.88 | -0.24 | 0.26 | 0.37 | 0.79 | 0.47 | 1.33 | 0.94 | -0.43 | 0.54 | 0.43 | 0.65 | 0.23 | 1.87 | 0.96 |
| JSW | genus LachnospiraceaeNK4A136group id 11319 | 28 | -0.12 | 0.19 | 0.52 | 0.88 | 0.61 | 1.29 | 0.88 | -0.24 | 0.27 | 0.37 | 0.78 | 0.46 | 1.33 | 0.94 | -0.55 | 0.52 | 0.30 | 0.58 | 0.21 | 1.59 | 0.96 |
| JSW | genus LachnospiraceaeUCG001 id 11321 | 28 | -0.24 | 0.25 | 0.34 | 0.79 | 0.49 | 1.28 | 0.88 | 0.06 | 0.34 | 0.87 | 1.06 | 0.54 | 2.07 | 0.96 | 0.15 | 0.62 | 0.81 | 1.17 | 0.35 | 3.91 | 0.96 |
| JSW | genus LachnospiraceaeUCG004 id 11324 | 28 | -0.30 | 0.23 | 0.19 | 0.74 | 0.47 | 1.16 | 0.88 | -0.35 | 0.31 | 0.26 | 0.71 | 0.39 | 1.29 | 0.94 | -0.19 | 0.56 | 0.73 | 0.83 | 0.28 | 2.46 | 0.96 |
| JSW | genus LachnospiraceaeUCG008 id 11328 | 27 | 0.47 | 0.30 | 0.12 | 1.60 | 0.88 | 2.88 | 0.88 | 0.91 | 0.43 | 0.03 | 2.48 | 1.07 | 5.76 | 0.94 | 1.02 | 0.80 | 0.21 | 2.78 | 0.58 | 13.41 | 0.96 |
| JSW | genus LachnospiraceaeUCG010 id 11330 | 28 | -0.09 | 0.22 | 0.66 | 0.91 | 0.59 | 1.40 | 0.88 | -0.08 | 0.30 | 0.79 | 0.92 | 0.52 | 1.65 | 0.94 | -0.13 | 0.59 | 0.83 | 0.88 | 0.27 | 2.82 | 0.96 |
| JSW | genus Lactobacillus id 1837 | 27 | 0.06 | 0.32 | 0.86 | 1.06 | 0.56 | 1.99 | 0.93 | 0.38 | 0.43 | 0.38 | 1.46 | 0.63 | 3.40 | 0.94 | 0.10 | 0.76 | 0.90 | 1.10 | 0.25 | 4.89 | 0.97 |
| JSW | genus Lactococcus id 1851 | 27 | -0.22 | 0.40 | 0.59 | 0.81 | 0.37 | 1.76 | 0.88 | -0.60 | 0.57 | 0.29 | 0.55 | 0.18 | 1.68 | 0.94 | -1.33 | 1.15 | 0.26 | 0.27 | 0.03 | 2.54 | 0.96 |
| JSW | genus Marvinbryantia id 2005 | 28 | -0.05 | 0.23 | 0.82 | 0.95 | 0.61 | 1.48 | 0.93 | -0.15 | 0.34 | 0.66 | 0.86 | 0.44 | 1.68 | 0.94 | -0.41 | 0.71 | 0.57 | 0.66 | 0.16 | 2.68 | 0.96 |
| JSW | genus Methanobrevibacter id 123 | 26 | 0.35 | 0.45 | 0.43 | 1.42 | 0.59 | 3.44 | 0.88 | -0.14 | 0.63 | 0.83 | 0.87 | 0.26 | 2.97 | 0.95 | -0.54 | 1.39 | 0.70 | 0.59 | 0.04 | 8.86 | 0.96 |
| JSW | genus Odoribacter id 952 | 28 | -0.04 | 0.22 | 0.85 | 0.96 | 0.62 | 1.48 | 0.93 | 0.29 | 0.30 | 0.32 | 1.34 | 0.75 | 2.40 | 0.94 | 0.60 | 0.48 | 0.22 | 1.82 | 0.71 | 4.62 | 0.96 |
| JSW | genus Olsenella id 822 | 27 | 0.44 | 0.46 | 0.33 | 1.56 | 0.63 | 3.81 | 0.88 | -0.32 | 0.59 | 0.59 | 0.73 | 0.23 | 2.33 | 0.94 | -1.37 | 1.20 | 0.26 | 0.25 | 0.02 | 2.67 | 0.96 |

| | | | | | | | | | | | | | | | | | | | | | | | |
|---|---|---|---|---|---|---|---|---|---|---|---|---|---|---|---|---|---|---|---|---|---|---|---|
| JSW | genus Oscillibacter id 2063 | 28 | 0.03 | 0.28 | 0.92 | 1.03 | 0.59 | 1.79 | 0.94 | -0.07 | 0.36 | 0.84 | 0.93 | 0.46 | 1.90 | 0.95 | -0.39 | 0.73 | 0.59 | 0.67 | 0.16 | 2.83 | 0.96 |
| JSW | genus Oscillospira id 2064 | 28 | 0.03 | 0.25 | 0.90 | 1.03 | 0.63 | 1.68 | 0.94 | 0.08 | 0.34 | 0.81 | 1.09 | 0.56 | 2.12 | 0.94 | 0.84 | 0.70 | 0.24 | 2.31 | 0.58 | 9.09 | 0.96 |
| JSW | genus Oxalobacter id 2978 | 27 | 0.11 | 0.38 | 0.78 | 1.11 | 0.52 | 2.36 | 0.92 | 0.26 | 0.52 | 0.61 | 1.30 | 0.47 | 3.58 | 0.94 | 1.13 | 1.14 | 0.33 | 3.08 | 0.33 | 28.77 | 0.96 |
| JSW | genus Parabacteroides id 954 | 28 | 0.09 | 0.19 | 0.65 | 1.09 | 0.75 | 1.59 | 0.88 | 0.12 | 0.28 | 0.67 | 1.13 | 0.66 | 1.93 | 0.94 | 0.86 | 0.57 | 0.14 | 2.37 | 0.77 | 7.24 | 0.96 |
| JSW | genus Paraprevotella id 962 | 28 | -0.09 | 0.31 | 0.78 | 0.92 | 0.50 | 1.67 | 0.92 | 0.22 | 0.41 | 0.60 | 1.24 | 0.55 | 2.79 | 0.94 | 0.57 | 0.80 | 0.48 | 1.77 | 0.37 | 8.42 | 0.96 |
| JSW | genus Parasutterella id 2892 | 28 | -0.23 | 0.24 | 0.32 | 0.79 | 0.50 | 1.26 | 0.88 | -0.24 | 0.32 | 0.46 | 0.79 | 0.42 | 1.48 | 0.94 | -0.20 | 0.66 | 0.77 | 0.82 | 0.22 | 3.00 | 0.96 |
| JSW | genus Peptococcus id 2037 | 27 | 0.51 | 0.39 | 0.19 | 1.67 | 0.77 | 3.61 | 0.88 | 0.39 | 0.49 | 0.43 | 1.47 | 0.57 | 3.81 | 0.94 | 0.26 | 1.05 | 0.81 | 1.30 | 0.16 | 10.19 | 0.96 |
| JSW | genus Phascolarctobacterium id 2168 | 28 | 0.15 | 0.24 | 0.53 | 1.16 | 0.73 | 1.86 | 0.88 | 0.36 | 0.33 | 0.27 | 1.44 | 0.76 | 2.73 | 0.94 | 0.70 | 0.59 | 0.25 | 2.02 | 0.63 | 6.43 | 0.96 |
| JSW | genus Prevotella7 id 11182 | 26 | 0.25 | 0.43 | 0.56 | 1.29 | 0.55 | 3.01 | 0.88 | 0.46 | 0.61 | 0.45 | 1.58 | 0.48 | 5.21 | 0.94 | 1.32 | 1.27 | 0.31 | 3.76 | 0.31 | 45.55 | 0.96 |
| JSW | genus Prevotella9 id 11183 | 28 | 0.03 | 0.33 | 0.93 | 1.03 | 0.54 | 1.98 | 0.94 | -0.28 | 0.38 | 0.46 | 0.75 | 0.36 | 1.59 | 0.94 | -0.71 | 0.79 | 0.37 | 0.49 | 0.10 | 2.30 | 0.96 |
| JSW | genus RikenellaceaeRC9gutgroup id 11191 | 26 | 0.46 | 0.44 | 0.31 | 1.58 | 0.66 | 3.77 | 0.88 | 0.09 | 0.60 | 0.89 | 1.09 | 0.34 | 3.53 | 0.96 | -0.27 | 1.18 | 0.82 | 0.77 | 0.08 | 7.81 | 0.96 |
| JSW | genus Romboutsia id 11347 | 28 | 0.20 | 0.21 | 0.33 | 1.23 | 0.81 | 1.85 | 0.88 | 0.28 | 0.30 | 0.34 | 1.33 | 0.74 | 2.38 | 0.94 | 0.42 | 0.61 | 0.49 | 1.53 | 0.46 | 5.03 | 0.96 |
| JSW | genus Roseburia id 2012 | 28 | -0.64 | 0.19 | 0.00 | 0.53 | 0.36 | 0.76 | 0.07 | -0.81 | 0.25 | 0.00 | 0.44 | 0.27 | 0.73 | 0.17 | -0.92 | 0.48 | 0.07 | 0.40 | 0.15 | 1.03 | 0.96 |
| JSW | genus Ruminiclostridium5 id 11355 | 28 | 0.05 | 0.19 | 0.80 | 1.05 | 0.72 | 1.52 | 0.93 | 0.19 | 0.26 | 0.47 | 1.21 | 0.72 | 2.03 | 0.94 | 0.31 | 0.57 | 0.59 | 1.36 | 0.45 | 4.13 | 0.96 |
| JSW | genus Ruminiclostridium6 id 11356 | 28 | 0.29 | 0.26 | 0.28 | 1.33 | 0.80 | 2.24 | 0.88 | 0.14 | 0.33 | 0.66 | 1.15 | 0.61 | 2.18 | 0.94 | -0.12 | 0.64 | 0.86 | 0.89 | 0.25 | 3.14 | 0.96 |
| JSW | genus Ruminiclostridium9 id 11357 | 28 | -0.11 | 0.19 | 0.58 | 0.90 | 0.61 | 1.31 | 0.88 | -0.28 | 0.26 | 0.29 | 0.76 | 0.45 | 1.27 | 0.94 | -0.50 | 0.49 | 0.32 | 0.61 | 0.23 | 1.60 | 0.96 |
| JSW | genus RuminococcaceaeNK4A214group id 11358 | 28 | 0.18 | 0.20 | 0.37 | 1.20 | 0.81 | 1.79 | 0.88 | 0.06 | 0.28 | 0.84 | 1.06 | 0.61 | 1.85 | 0.95 | -0.29 | 0.55 | 0.61 | 0.75 | 0.26 | 2.20 | 0.96 |
| JSW | genus RuminococcaceaeUCG002 id 11360 | 28 | -0.07 | 0.19 | 0.73 | 0.93 | 0.64 | 1.37 | 0.91 | -0.07 | 0.27 | 0.79 | 0.93 | 0.55 | 1.57 | 0.94 | -0.06 | 0.63 | 0.92 | 0.94 | 0.27 | 3.25 | 0.98 |
| JSW | genus RuminococcaceaeUCG003 id 11361 | 28 | 0.01 | 0.21 | 0.97 | 1.01 | 0.67 | 1.52 | 0.97 | -0.24 | 0.30 | 0.43 | 0.79 | 0.44 | 1.42 | 0.94 | -0.25 | 0.66 | 0.71 | 0.78 | 0.22 | 2.83 | 0.96 |

| | | | | | | | | | | | | | | | | | | | | | | | |
|---|---|---|---|---|---|---|---|---|---|---|---|---|---|---|---|---|---|---|---|---|---|---|---|
| JSW | genus RuminococcaceaeUCG004 id 11362 | 28 | -0.05 | 0.26 | 0.84 | 0.95 | 0.57 | 1.59 | 0.93 | 0.10 | 0.34 | 0.78 | 1.10 | 0.56 | 2.16 | 0.94 | 0.09 | 0.68 | 0.90 | 1.09 | 0.29 | 4.17 | 0.97 |
| JSW | genus RuminococcaceaeUCG005 id 11363 | 28 | 0.18 | 0.20 | 0.35 | 1.20 | 0.82 | 1.77 | 0.88 | 0.23 | 0.27 | 0.40 | 1.25 | 0.74 | 2.11 | 0.94 | 0.51 | 0.53 | 0.34 | 1.66 | 0.59 | 4.66 | 0.96 |
| JSW | genus RuminococcaceaeUCG009 id 11366 | 27 | 0.20 | 0.31 | 0.52 | 1.22 | 0.67 | 2.23 | 0.88 | -0.07 | 0.43 | 0.88 | 0.94 | 0.40 | 2.17 | 0.96 | -0.33 | 0.88 | 0.71 | 0.72 | 0.13 | 4.02 | 0.96 |
| JSW | genus RuminococcaceaeUCG010 id 11367 | 28 | -0.01 | 0.23 | 0.96 | 0.99 | 0.63 | 1.56 | 0.96 | -0.10 | 0.31 | 0.74 | 0.90 | 0.49 | 1.65 | 0.94 | -0.04 | 0.65 | 0.95 | 0.96 | 0.27 | 3.43 | 0.98 |
| JSW | genus RuminococcaceaeUCG011 id 11368 | 27 | 0.23 | 0.48 | 0.63 | 1.26 | 0.49 | 3.22 | 0.88 | -0.60 | 0.61 | 0.32 | 0.55 | 0.17 | 1.80 | 0.94 | -1.10 | 1.36 | 0.42 | 0.33 | 0.02 | 4.78 | 0.96 |
| JSW | genus RuminococcaceaeUCG013 id 11370 | 28 | 0.12 | 0.21 | 0.56 | 1.13 | 0.75 | 1.70 | 0.88 | 0.13 | 0.28 | 0.65 | 1.13 | 0.65 | 1.97 | 0.94 | 0.10 | 0.51 | 0.85 | 1.10 | 0.40 | 3.03 | 0.96 |
| JSW | genus RuminococcaceaeUCG014 id 11371 | 21 | 0.13 | 0.29 | 0.65 | 1.14 | 0.65 | 2.00 | 0.88 | -0.25 | 0.35 | 0.46 | 0.78 | 0.39 | 1.53 | 0.94 | -0.23 | 0.75 | 0.77 | 0.80 | 0.19 | 3.44 | 0.96 |
| JSW | genus Ruminococcus1 id 11373 | 28 | -0.24 | 0.19 | 0.21 | 0.78 | 0.54 | 1.15 | 0.88 | -0.36 | 0.27 | 0.17 | 0.70 | 0.41 | 1.17 | 0.94 | -0.97 | 0.63 | 0.14 | 0.38 | 0.11 | 1.31 | 0.96 |
| JSW | genus Ruminococcus2 id 11374 | 28 | 0.45 | 0.21 | 0.04 | 1.56 | 1.03 | 2.38 | 0.88 | 0.36 | 0.29 | 0.21 | 1.44 | 0.82 | 2.52 | 0.94 | 0.63 | 0.57 | 0.27 | 1.88 | 0.62 | 5.73 | 0.96 |
| JSW | genus Sellimonas id 14369 | 26 | -0.53 | 0.45 | 0.25 | 0.59 | 0.24 | 1.44 | 0.88 | 0.17 | 0.63 | 0.79 | 1.19 | 0.34 | 4.09 | 0.94 | 0.89 | 1.40 | 0.53 | 2.44 | 0.16 | 37.65 | 0.96 |
| JSW | genus Senegalimassilia id 11160 | 28 | 0.14 | 0.32 | 0.66 | 1.15 | 0.61 | 2.15 | 0.88 | 0.28 | 0.43 | 0.52 | 1.32 | 0.57 | 3.07 | 0.94 | 0.85 | 0.93 | 0.37 | 2.33 | 0.38 | 14.35 | 0.96 |
| JSW | genus Slackia id 825 | 27 | -0.08 | 0.32 | 0.81 | 0.93 | 0.49 | 1.74 | 0.93 | 0.56 | 0.45 | 0.21 | 1.76 | 0.72 | 4.27 | 0.94 | 0.74 | 0.95 | 0.44 | 2.09 | 0.33 | 13.43 | 0.96 |
| JSW | genus Streptococcus id 1853 | 28 | -0.09 | 0.20 | 0.66 | 0.92 | 0.62 | 1.35 | 0.88 | -0.08 | 0.28 | 0.76 | 0.92 | 0.54 | 1.58 | 0.94 | -0.05 | 0.58 | 0.94 | 0.96 | 0.30 | 3.00 | 0.98 |
| JSW | genus Subdoligranulum id 2070 | 28 | 0.24 | 0.25 | 0.33 | 1.28 | 0.78 | 2.08 | 0.88 | 0.03 | 0.30 | 0.91 | 1.03 | 0.58 | 1.84 | 0.97 | 1.23 | 0.72 | 0.10 | 3.41 | 0.83 | 14.05 | 0.96 |
| JSW | genus Sutterella id 2896 | 28 | -0.18 | 0.22 | 0.42 | 0.84 | 0.55 | 1.29 | 0.88 | -0.19 | 0.30 | 0.54 | 0.83 | 0.46 | 1.50 | 0.94 | -0.14 | 0.60 | 0.81 | 0.87 | 0.27 | 2.79 | 0.96 |
| JSW | genus Terrisporobacter id 11348 | 28 | 0.13 | 0.35 | 0.71 | 1.14 | 0.57 | 2.27 | 0.91 | -0.40 | 0.45 | 0.37 | 0.67 | 0.28 | 1.62 | 0.94 | -0.99 | 0.78 | 0.22 | 0.37 | 0.08 | 1.72 | 0.96 |
| JSW | genus Turicibacter id 2162 | 28 | 0.17 | 0.28 | 0.55 | 1.19 | 0.68 | 2.07 | 0.88 | 0.29 | 0.39 | 0.46 | 1.33 | 0.63 | 2.83 | 0.94 | 0.48 | 0.73 | 0.51 | 1.62 | 0.39 | 6.77 | 0.96 |
| JSW | genus Tyzzerella3 id 11335 | 27 | 0.24 | 0.35 | 0.48 | 1.28 | 0.65 | 2.52 | 0.88 | 0.48 | 0.48 | 0.32 | 1.61 | 0.63 | 4.10 | 0.94 | 1.33 | 1.03 | 0.21 | 3.77 | 0.50 | 28.65 | 0.96 |
| JSW | genus Veillonella id 2198 | 27 | -0.48 | 0.31 | 0.12 | 0.62 | 0.34 | 1.13 | 0.88 | -0.51 | 0.38 | 0.18 | 0.60 | 0.28 | 1.27 | 0.94 | -0.91 | 0.71 | 0.21 | 0.40 | 0.10 | 1.63 | 0.96 |

| | | | | | | | | | | | | | | | | | | | | | | |
|---|---|---|---|---|---|---|---|---|---|---|---|---|---|---|---|---|---|---|---|---|---|---|
| JSW | genus Victivallis id 2256 | 22 | 0.23 | 0.48 | 0.63 | 1.26 | 0.49 | 3.24 | 0.88 | -0.32 | 0.69 | 0.64 | 0.72 | 0.19 | 2.81 | 0.94 | -1.23 | 1.35 | 0.37 | 0.29 | 0.02 | 4.16 | 0.96 |

JSW: jobs invovling shift work

**Table S7.** Sensitivity analysis of the MR analysis of the causal effect of JSW on gut microbiota.

| Exposure | Outcome | nSNP | Horizontal pleiotropy (MR-Egger) | | | Heterogeneity （Cochran's Q test) | | | | MR-PRESSO |
|---|---|---|---|---|---|---|---|---|---|---|
| | | | Egger intercept | SE | p | Q_MR_Egger | p | Q_IVW | p | p |
| JSW | phylum Actinobacteria | 28 | -0.002796196 | 0.013912626 | 0.84 | 28.51573226 | 0.33 | 28.56003 | 0.38 | 0.38 |
| JSW | class Coriobacteriia | 28 | -0.019893025 | 0.013384701 | 0.15 | 25.15848073 | 0.51 | 27.36742 | 0.44 | 0.44 |
| JSW | order Coriobacteriales | 28 | -0.019893025 | 0.013384701 | 0.15 | 25.15848073 | 0.51 | 27.36742 | 0.44 | 0.46 |
| JSW | family Coriobacteriaceae | 28 | -0.019893025 | 0.013384701 | 0.15 | 25.15848073 | 0.51 | 27.36742 | 0.44 | 0.46 |
| JSW | genus *Eubacteriumbrachy* group | 27 | -0.019296882 | 0.029801414 | 0.52 | 18.99217137 | 0.80 | 19.41145 | 0.82 | 0.81 |
| JSW | genus *Ruminococcusgnavus* group | 27 | 0.005017939 | 0.02721106 | 0.86 | 33.25543714 | 0.12 | 33.30067 | 0.15 | 0.16 |
| JSW | genus *Lachnoclostridium* | 28 | 0.008625679 | 0.013310645 | 0.52 | 21.64226319 | 0.71 | 22.0622 | 0.73 | 0.76 |
| JSW | genus *Roseburia* | 28 | -0.02289201 | 0.013327094 | 0.10 | 12.57190291 | 0.99 | 15.52241 | 0.96 | 0.97 |
| JSW | genus *Ruminococcus2* | 28 | 0.04630657 | 0.014354722 | 0.00 | 19.59460449 | 0.81 | 30.00089 | 0.31 | 0.34 |

JSW: jobs invovling shift work

**Figure S1.** Scatter plots and leave-one-out stability test results of the causal estimates of exposure (JSW) on outcomes (coronary heart disease, diabetes, and hypertension). **Panel A.** Scatter plot of the causal estimates of exposure (JSW) on coronary heart disease; **Panel B.** scatter plot of the causal estimates of exposure (JSW) on type 2 diabetes; **Panel C.** scatter plot of the causal estimates of exposure (JSW) on hypertension); **Panel D.** leave-one-out stability test results for the causal estimates of exposure (JSW) on coronary heart disease; **Panel E.** Leave-one-out stability test results for the causal estimates of exposure (JSW) on type 2 diabetes; **Panel F.** Leave-one-out stability test results for the causal estimates of exposure (JSW) on hypertension. JSW: jobs involving shift work.

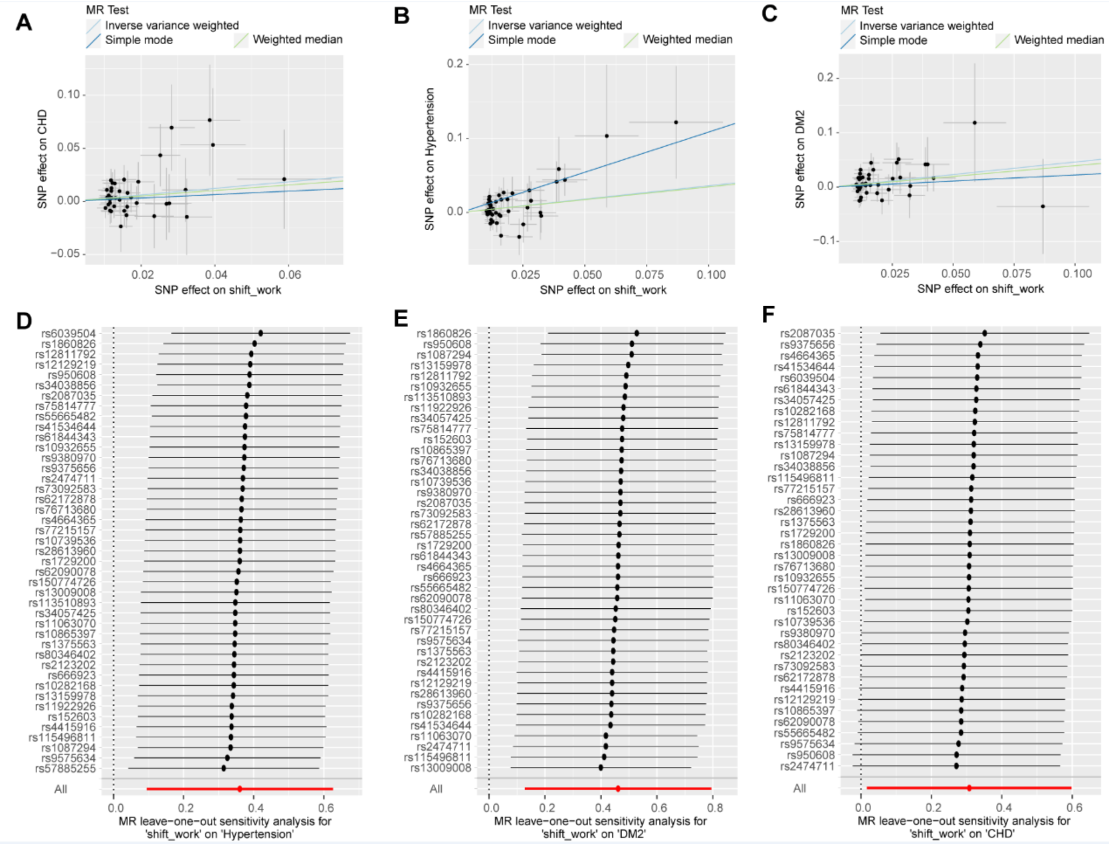

**Figure S2.** Scatter plots and leave-one-out stability test results for the causal estimates of exposure (JSW) on gut microbiota. **Panel A-I.**Scatter plots of the causal estimates of exposure (JSW) on nine significant gut microbiota taxa; **Panel J-R.** leave-one-out stability test results for the causal estimates of exposure (JSW) on the nine significant gut microbiota taxa. JSW: jobs involving shift work.

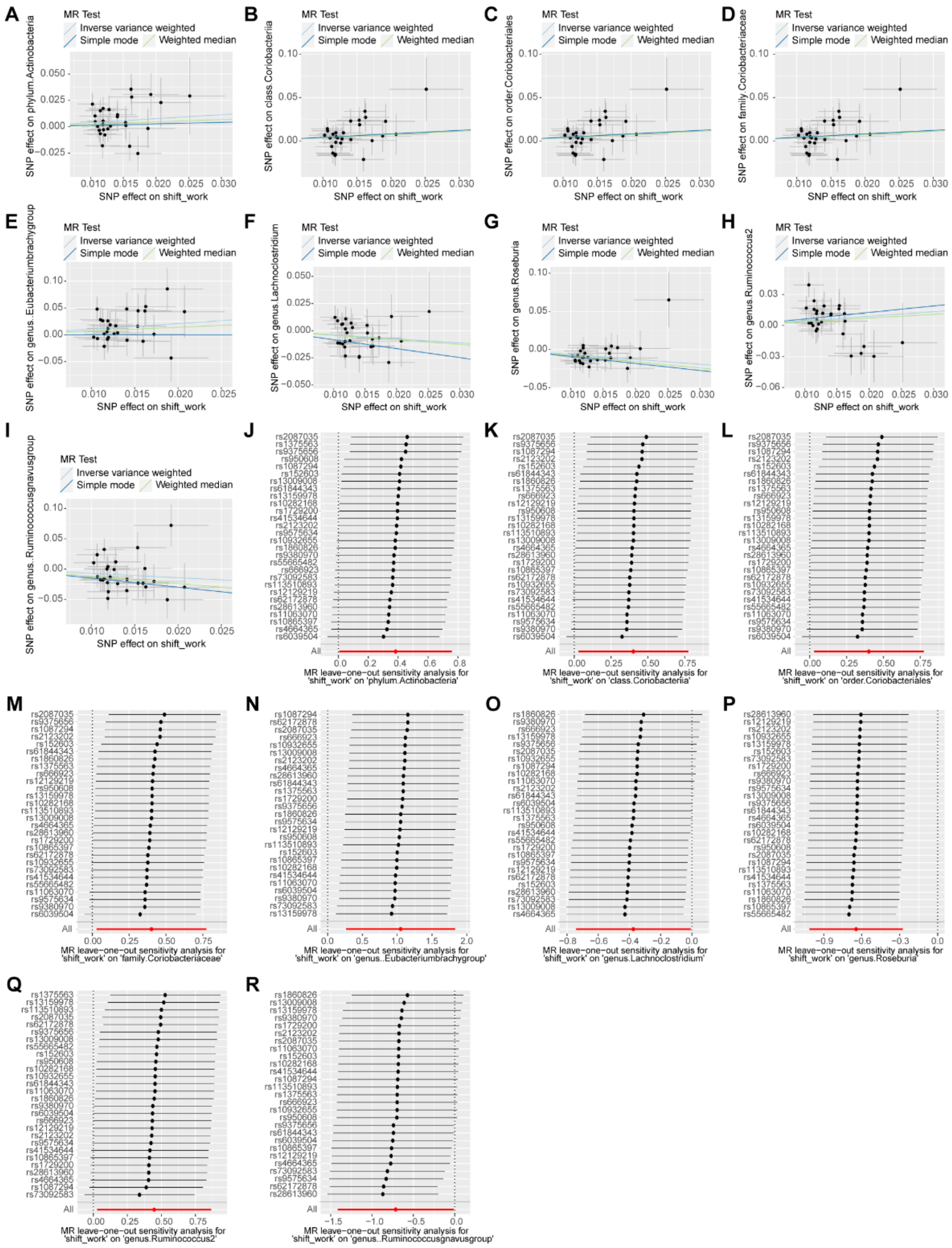

Supplement: Online Supplementary Document [file jogh-14-04110-s001.pdf]
